# Mass transport-dependent *in situ* Raman detection in CO/CO_2_ electrolysis

**DOI:** 10.1039/d5sc07260c

**Published:** 2025-12-16

**Authors:** Wen Yan, Hangyu Bu, Xinjuan Du, Beining Xu, Jia Liu, Ming Ma

**Affiliations:** a School of Chemical Engineering and Technology, Xi'an Jiaotong University Xi'an 710049 People's Republic of China mingma@xjtu.edu.cn; b Instrument Analysis Center, Xi'an Jiaotong University Xi'an 710049 People's Republic of China liujia6j@xjtu.edu.cn

## Abstract

*In situ* Raman spectroscopy has been widely employed in the CO_2_/CO electroreduction field to extract mechanistic insights into reaction pathways toward product formation. However, most of the previous *in situ* Raman studies are based on H-type spectroelectrochemical cells, which constrain the mass transport to the catalyst surface, potentially affecting the coverage of key intermediates relevant to the Raman signals and even distorting the mechanistic understanding. Here, we present a systematic comparison for the *in situ* Raman detection of intermediates during CO_2_/CO reduction between an H-type spectroelectrochemical cell and a GDE-type spectroelectrochemical flow cell. We found that cell configurations exert a minimal influence on the *in situ* Raman detection and analysis of surface-adsorbed intermediates during CO_2_ reduction, which is likely linked to the high solubility of CO_2_ in aqueous media. In contrast, during CO reduction, the severe CO mass transport limitations in H-type spectroelectrochemical cells significantly lower the formation and coverage of key intermediates, resulting in *in situ* Raman detection and analysis results that are distinct from those obtained using GDE-type spectroelectrochemical flow cells. Thereby, circumventing mass transport limitation is crucial for *in situ* Raman tests to unveil the underlying mechanism of electrolysis.

## Abstract 2

Circumventing mass transport limitation is essential for *in situ* Raman tests to unveil the underlying mechanism of CO electrolysis.

## Graphical abstract

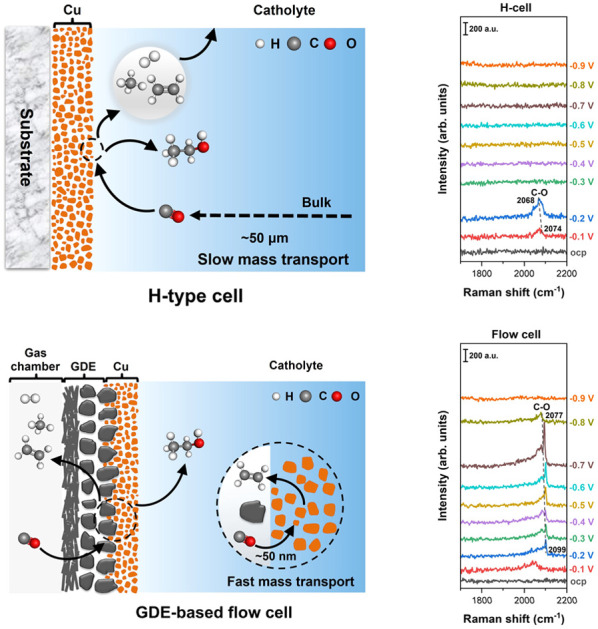

## Introduction

Electrocatalytic CO_2_/CO reduction to high-value fuels and chemicals is widely recognized as a promising strategy for achieving a sustainable carbon cycle.^1–4^ To date, Cu remains the only monometallic catalyst that is capable of directly converting CO_2_/CO into valuable oxygenates and hydrocarbons.^5,6^ However, its limited selectivity toward specific multi-carbon (C_2+_) product at commercially-relevant current densities poses a significant challenge for practical applications.^7,8^ Previous studies have shown that the dynamic evolution of *CO intermediates on the Cu catalyst surface, including adsorption configurations,^9–11^ surface coverage,^12,13^ and C–C coupling pathways,^14,15^ is crucial in governing the selectivity for C_2+_ products. To better understand the reaction mechanism of C_2+_ product formation and achieve controllable C_2+_ selectivity, many attempts have focused on the detection of the transient evolution of reaction key intermediates and the dynamic restructuring of active sites during electrocatalysis.^16^ In this context, *in situ* spectroscopic techniques are urgently needed to monitor the microscopic interfacial processes at the catalyst surface in real-time.^9,17–22^

*In situ* infrared (IR) and Raman spectroscopy, which are two widely employed techniques for probing molecular vibrational characteristics, have been extensively used to investigate the dynamic behavior of surface species and reaction mechanisms in electrocatalysis.^18,20,23–30^ Among the two techniques, *in situ* IR spectroscopy with its high signal-to-noise ratio and excellent temporal resolution can precisely track the dynamic evolution of reaction intermediates,^18,25,27,28,30^ but its low sensitivity in the low-wavenumber region significantly restricts the detection of characteristic vibrational signals associated with key intermediates, such as adsorbed CO and metal–carbon bonds.^10,31,32^ In contrast, *in situ* Raman spectroscopy is capable of overcoming this limitation owing to its high sensitivity to low-wavenumber vibrations, and it has been broadly applied to probe the key intermediates and reaction paths associated with the formation of final products and to gain a better understanding of the interfacial reaction processes during CO_2_/CO electrolysis.^23,24,29,33^

In recent years, the CO_2_/CO electrolysis field has progressed from H-cells to flow electrolyzers with gas diffusion electrodes (GDEs).^14,34–36^ However, to the best of our knowledge, the majority of *in situ* Raman studies have still been performed in H-type spectroelectrochemical cells.^23,24,32,33,37–39^ The thick mass transfer boundary layer in H-type spectroelectrochemical cells significantly restricts reactant (*i.e.* CO_2_/CO) transport to the catalyst surface, which may influence the intermediates coverage that is linked to the signal for *in situ* Raman spectroscopy experiments,^40^ particularly in the case of CO reduction due to the extremely low CO solubility in electrolytes.^36^ Thereby, the poor mass transport of reactants in traditional H-type spectroelectrochemical cells may inadvertently influence the detection of key intermediates and the related mechanistic analysis. To circumvent mass transport limitations and get better mechanistic insights into the formation and coverage of key intermediates, GDE-type spectroelectrochemical flow cells should be employed for the measurement of *in situ* Raman spectroscopy. However, the specific discrepancy in the *in situ* Raman detection of CO_2_/CO reduction intermediates between H-type spectroelectrochemical cells and GDE-type spectroelectrochemical flow cells remains unclear.

Herein, we demonstrate a systematic comparison for the *in situ* Raman detection of intermediates in CO_2_/CO electrolysis with and without mass transport limitations. We found that although cell configurations do not play an important role in the *in situ* Raman detection and analysis of surface-adsorbed intermediates in CO_2_ reduction, CO electrolysis in commonly used H-type spectroelectrochemical cells provided *in situ* Raman detection and analysis results that are significantly distinct from those acquired in GDE-type spectroelectrochemical flow cells, which include intermediates related to C_2+_ products. Further analysis reveals that the CO mass transport constraints during CO reduction in H-type spectroelectrochemical cells significantly reduce the formation and coverage of key intermediates, inadvertently influencing *in situ* Raman detection results.

## Results and discussion

### Catalyst characterization and *in situ* Raman tests

**Fig. 1:**
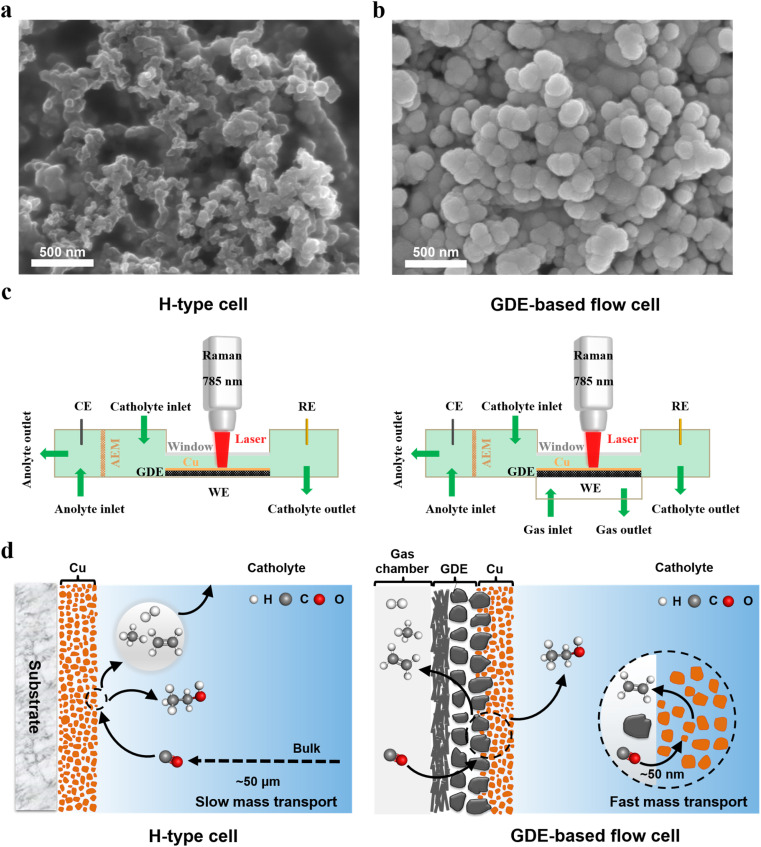
Morphology characterization and cell design. SEM images of (a) microporous layers of a GDE (Sigracet 39 BB) and (b) Cu catalyst layers coated on a GDE. (c) Schematic illustrations of an H-type spectroelectrochemical cell (left) and a GDE-type spectroelectrochemical flow cell (right) for *in situ* Raman spectroscopy. (d) Comparison of mass transport in two representative spectroelectrochemical configurations: an H-type cell (left) and a GDE-based flow cell (right). The thickness of the mass-transport boundary layer in the two cells was obtained from ref. 34 and 42.

The Cu catalysts were deposited on the microporous layers of GDEs (Fig. 1a) using direct current magnetron sputtering at an argon pressure of 0.5 Pa (Fig. S1). The vacuum deposition technique enables the fabrication of catalyst layers with high purity and excellent reproducibility. Fig. 1b shows that the deposited Cu electrocatalyst had a rough surface, consisting of densely packed Cu nanoparticles with an average diameter of ∼100 nm. The rough surface morphology can significantly enhance the local electromagnetic field, thereby inducing a surface-enhanced effect that substantially amplifies the intensity of the *in situ* Raman signal when using the incident light with a wavelength of 785 nm.^33,37^ Additionally, all catalysts used in this study were Cu films with a uniform thickness of approximately 200 nm (Fig. S2).

To explore the discrepancy in the *in situ* Raman detection of CO_2_/CO reduction intermediates with and without mass transport limitations, *in situ* Raman spectroscopy was performed using two spectroelectrochemical cell configurations (Fig. 1c, S4 and S5) with distinct mass transport characteristics: an H-type spectroelectrochemical cell and a GDE-based spectroelectrochemical flow cell. Specifically, the conventional H-type spectroelectrochemical cell consists of two compartments (the left scheme of Fig. 1c), namely the catholyte and anolyte compartments, which are separated by an anion exchange membrane (AEM). During *in situ* Raman experiments, CO/CO_2_ saturated electrolytes were continuously circulated through both the catholyte and anolyte compartments using two peristaltic pumps. The extremely thick diffusion layer for gas reactants in H-type spectroelectrochemical cells leads to significantly sluggish mass transport in the electrolyte (the left scheme of Fig. 1d).^34,41^ In contrast, a custom-designed spectroelectrochemical flow cell with a GDE, where the mass-transport of gas reactant can be accelerated significantly, was employed for *in situ* Raman experiments. As shown in the right scheme of Fig. 1c, the GDE-based spectroelectrochemical flow cell is composed of a catholyte and an anolyte chamber, separated by an AEM, and a gas chamber that allows gas flow into and out of the flow cell during electrolysis. The gas diffusion layer of the GDE, positioned between catholyte and gas chambers, allows gas reactants to access the catalyst surface *via* an extremely short diffusion layer (the right scheme of Fig. 1d).^34,41^

Additionally, all the CO_2_/CO reduction tests in both cells were based on a three-electrode configuration, in which a GDE loaded with Cu catalysts served as the working electrode, while an Ag/AgCl or Hg/HgO electrode and a graphite rod were used as the reference electrode and the counter electrode, respectively (Fig. 1c). To ensure reliable comparison of peak areas in the *in situ* Raman detection, all of the Raman spectra were acquired on the identical cathodic GDE under a constant electrolyte flow rate (14 mL min^−1^).

### *In situ* Raman spectroscopy measurements during electrochemical CO_2_ reduction

To explore the impact of mass transport on the analysis of species adsorbed on Cu surfaces during electrocatalytic CO_2_ reduction, we performed *in situ* Raman spectroscopy in an H-type spectroelectrochemical cell and a GDE-type spectroelectrochemical flow cell, respectively. Additionally, it should be noted that when conducting CO_2_ electrolysis in GDE-type flow electrolyzers, carbonate formation near the cathodic GDE/catholyte interface could lead to a rapid variation in the composition, concentration and pH of the electrolyte for most commonly used electrolytes.^35,42,43^ The electrolyte variation over electrolysis may influence the formation and coverage of intermediates related to CO_2_ reduction. Herein, in order to circumvent the electrolyte variation over electrolysis and to readily compare the difference *in situ* Raman detection caused by the two distinct cell configurations, all the CO_2_ tests were carried out in 0.5 M CO_2_-saturated KHCO_3_ electrolyte (the ionic species and pH of the electrolyte can be maintained using a CO_2_-saturated bicarbonate electrolyte^36,43^).

**Fig. 2:**
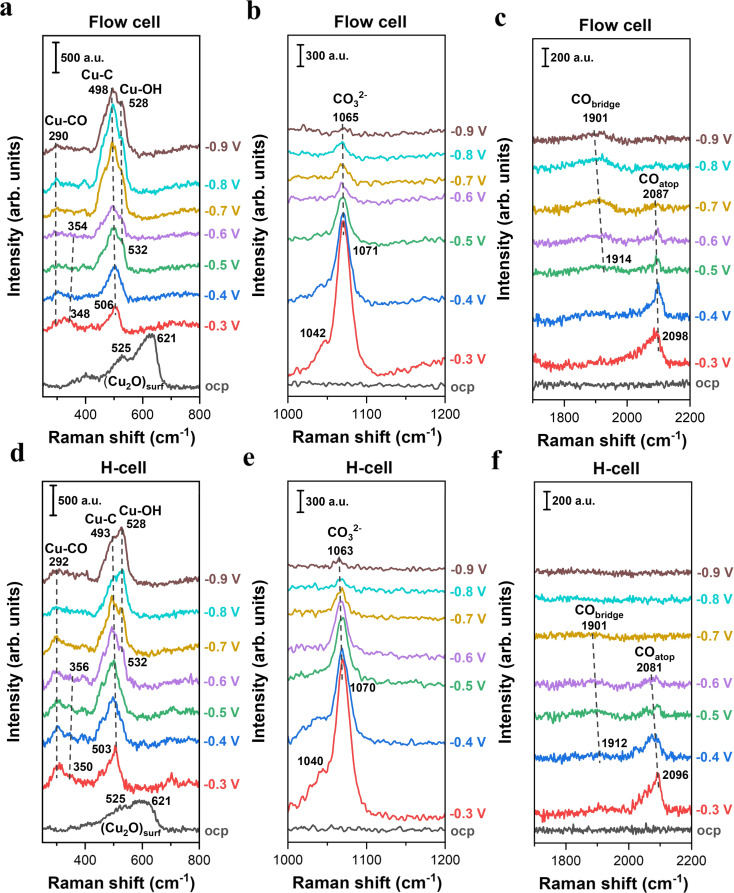
*In situ* Raman spectroscopy of electrochemical CO_2_ reduction on Cu catalysts in 0.5 M KHCO_3_ using two representative *in situ* Raman configurations. (a–c) Raman spectra on Cu catalysts as a function of applied potentials (*vs.* RHE, no *iR* correction) collected in the GDE-type spectroelectrochemical flow cell. (d–f) Raman spectra on Cu catalysts as a function of applied potentials (*vs.* RHE, no *iR* correction) collected in the H-type spectroelectrochemical cell.

Fig. 2 presents a comparison of the *in situ* Raman spectra of surface species on Cu catalysts obtained from two spectroelectrochemical configurations with distinct mass transport conditions. In the low-frequency region, two characteristic peaks at 525 and 621 cm^−1^ attributed to surface Cu_2_O were observed in both cell configurations at the open-circuit potential (OCP),^23,24^ indicating the presence of a native oxide layer on Cu surfaces upon exposure to ambient air (Fig. 2a, d and S7). Once applying negative potentials (even as low as −0.3 V), the Raman peaks associated with the Cu_2_O layer disappeared, which is due to the electroreduction of Cu oxides to metallic Cu.^44^ This result suggests that all the *in situ* Raman spectra in this work were acquired on the metallic Cu surface at negative potentials.

When using the GDE-type spectroelectrochemical flow cell, the broad band centered at ∼506 cm^−1^ (Fig. 2a) was observed in the low-frequency region under an applied potential of −0.3 V, which is attributed to Cu–C related intermediates.^45–47^ Regarding the Cu–C peak, there are two different theories: (i) the Cu–C peak reflects the complex C-related intermediates during CO_2_ conversion on the Cu surface;^45^ (ii) the Cu–C peak is likely correlated with the surface coverage of bridge-bonded CO (CO_bridge_) at ∼1914 cm^−1^ (Fig. 2c).^46,47^ In this study, we found that the integrated area of the Cu–C peak initially increased when lowering the potentials from −0.3 V to −0.8 V, and subsequently diminished at more negative potentials than −0.8 V (Fig. S8). This trend directly follows the variation in the CO_bridge_ peak intensity as a function of potential (Fig. S9), which may suggest that the Cu–C peak is likely linked to the coverage of CO_bridge_. As a comparison, we also found an obvious Cu–C peak under the use of an H-type spectroelectrochemical cell (Fig. 2d). Additionally, the variation trend of the Cu–C peak area as a function of potential also follows CO_bridge_ peak area in the H-cell (Fig. S9). These findings reveal that while there is a debate regarding the Cu–C peak in the field, both cells can provide the similar detection results of *in situ* Raman for the Cu–C peak and CO_bridge_ when performing CO_2_ electroreduction.

**Fig. 3:**
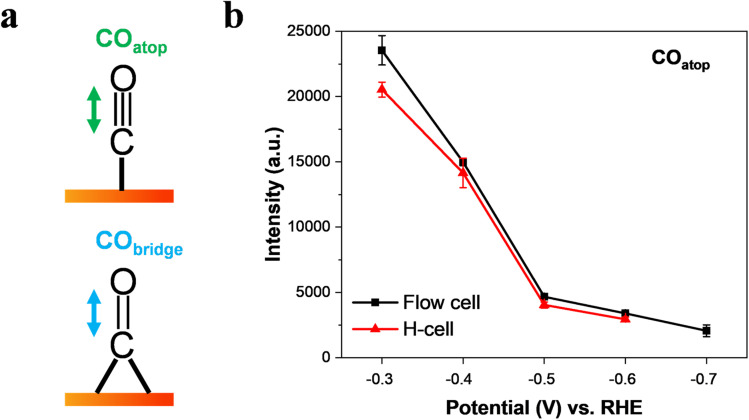
Intensity of the C≡O stretch band of CO_atop_ as a function of applied potential in CO_2_-saturated 0.5 M KHCO_3_. (a) Schematic view of the C≡O stretching mode of CO_atop_ and CO_bridge_ on a Cu surface. (b) Effect of potential on the intensity of the CO_atop_ band on Cu catalysts collected in a GDE-type spectroelectrochemical flow cell and an H-type spectroelectrochemical cell. The data were obtained by Gaussian peak fitting of the Raman spectra from Fig. 2c and f. The error bars represent the standard deviation from at least three independent measurements.

It is well-known that CO is the key intermediate in the formation of hydrocarbons and oxygenates on the Cu surface.^14,36,48^ As expected, we found that the C–O stretching peak from 2098 cm^−1^ to ∼2096 cm^−1^ in the flow-cell (Fig. 2c) and the H-cell (Fig. 2f) at −0.3 V corresponds to adsorbed CO in the atop configuration (CO_atop_).^10,36^ Meanwhile, the Cu–CO rotation (∼290 cm^−1^) and the Cu–C stretching (∼350 cm^−1^) features were also detected in both cell configurations (Fig. 2a and d). Importantly, it has been demonstrated that the CO_atop_ (Fig. 3a) contributes to the further conversion of CO intermediates into hydrocarbons.^10,31^ Thus, to uncover the mass transport effect, it is essential to compare the coverage of CO_atop_ during CO_2_ electroreduction between the flow-cell and the H-cell. Previous work has indicated that the integrated band area is directly proportional to the CO coverage on the Cu surface.^9,49^ Thus, to better illustrate the CO_atop_ coverage in the two cases, the peak area of the CO_atop_ at various potentials was quantified by integrating the Gaussian-fitted Raman spectra (Fig. 3b). As shown in Fig. 3b, we found the roughly equal integral area of the CO_atop_ peak between the H-type spectroelectrochemical cell and the GDE-type spectroelectrochemical flow cell when applying a fixed potential. This observation implies that the cell configuration has a minimal effect on CO_atop_ coverage during CO_2_ reduction.

In addition to the intermediates related to CO_2_ reduction products, the symmetric stretching mode of the C–O bond in carbonate was observed as a strong band of ∼1071 cm^−1^ in both cell configurations (Fig. 2b and e) under relatively less negative potentials. The carbonate band redshifted from 1071 to 1065 cm^−1^ as the applied potential decreased from −0.3 to −0.9 V. This redshift corresponds to a Stark tuning rate of 10 cm^−1^ V^−1^ (Fig. S10), which is in reasonable agreement with the previous work (12 cm^−1^ V^−1^).^33^ Additionally, the Cu–OH stretching mode at ∼532 cm^−1^ (Fig. 2a and d) suggests the presence of surface-adsorbed OH species.^47,50^

Based on the above results in this section, we therefore conclude that cell configurations could not significantly affect the *in situ* Raman detection and analysis of surface-adsorbed intermediates during CO_2_ electroreduction, and even an H-type spectroelectrochemical cell should be able to provide a reliable detection of *in situ* Raman for CO_2_ reduction, particularly for the relatively low reaction rates. This conclusion is likely linked to the relatively high solubility of CO_2_ in aqueous media and relatively low reaction rates.^51^

### *In situ* Raman spectroscopy measurements during electrochemical CO reduction

CO_2_ generates carbonate or bicarbonate through a buffering reaction in alkaline electrolytes, resulting in substantial alterations in ionic composition and pH of the electrolyte.^35,42,43^ In contrast, CO does not participate in buffering reactions with OH^−^ and thus does not affect the ionic composition or pH of the electrolyte. Additionally, CO serves as a key intermediate for C–C coupling toward C_2+_ products. Thereby, CO reduction has been widely utilized as a significant method for gaining mechanistic insights into the formation of C_2+_ products.^14,36,48^ In recent years, to get better understanding of the reaction mechanisms of C_2+_ product formation, adsorbed surface species on Cu surfaces from CO reduction have been intensively explored *via in situ* Raman spectroscopy.^24,31,36–39,52^ However, to the best of our knowledge, all of the previous *in situ* Raman studies based on CO reduction have been operated in H-type spectroelectrochemical cells (with the one exception of CO reduction^36^).^24,31,37–39,52^ The extremely low solubility of CO in electrolytes further exacerbates the CO mass transport limitations in H-type spectroelectrochemical cells,^36^ which severely constrains CO supply to the catalyst surface, significantly influencing the formation and coverage of intermediates that lead to reaction pathways toward final products. The variation in the coverage of intermediates is closely correlated to the detection and analysis of Raman signals during CO reduction, which may inevitably affect the understanding of the underlying reaction mechanisms.

**Fig. 4:**
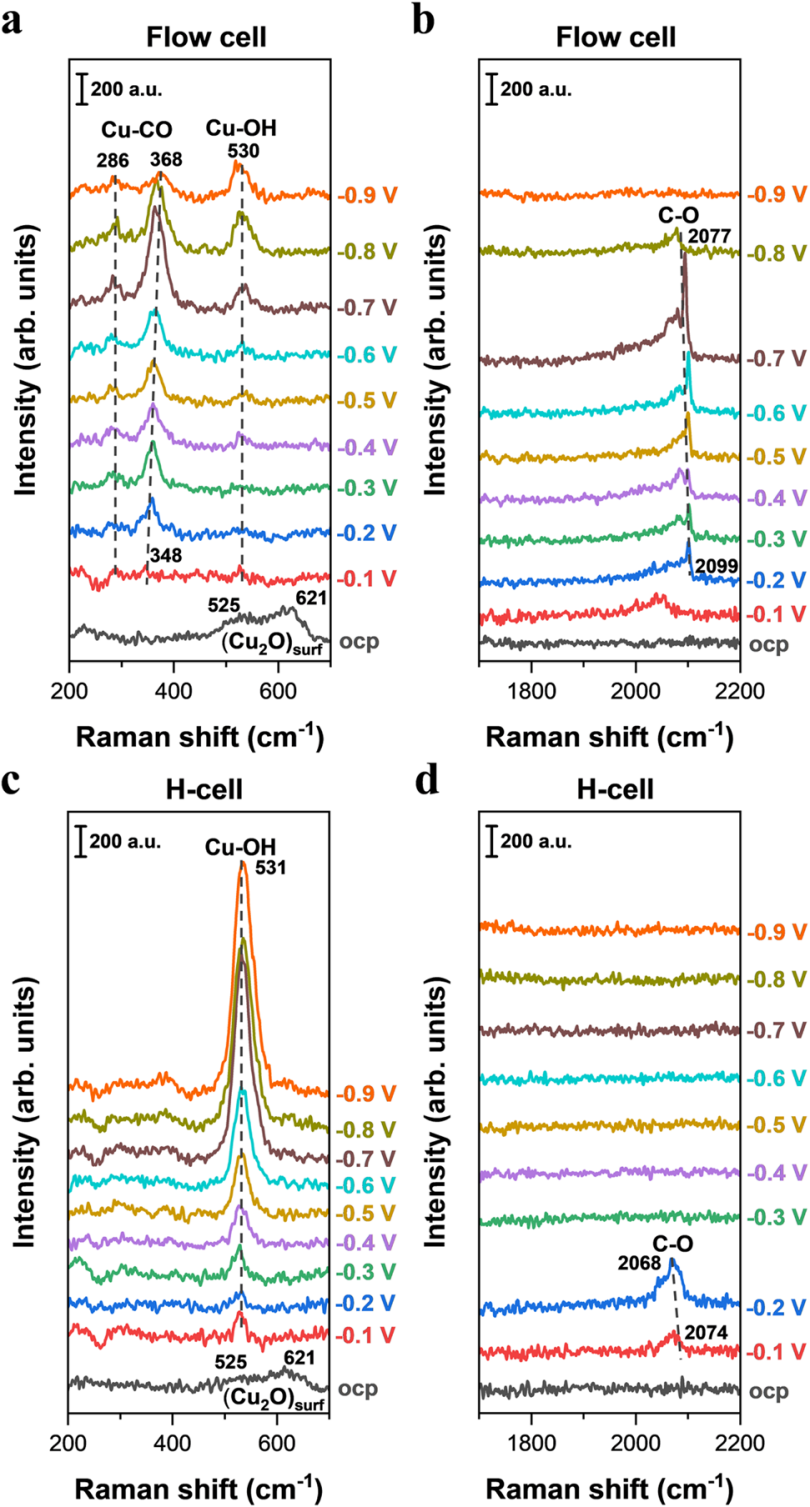
*In situ* Raman spectroscopy of electrochemical CO reduction on Cu catalysts in 0.5 M KOH using two representative *in situ* Raman configurations. (a and b) Raman spectra on Cu catalysts as a function of applied potentials (*vs.* RHE, no *iR* correction) collected in the GDE-type spectroelectrochemical flow cell. (c and d) Raman spectra on Cu catalysts as a function of applied potentials (*vs.* RHE, no *iR* correction) collected in the H-type spectroelectrochemical cell.

To uncover the influence of mass transport on the spectral signals of surface species during electrocatalytic CO reduction, we conducted *in situ* Raman spectroscopy on Cu catalysts in an H-type spectroelectrochemical cell and a GDE-type spectroelectrochemical cell, respectively. Here, in order to circumvent the effect of electrolyte variation and to maintain the ionic species and pH of electrolyte over electrolysis,^43^ 0.5 M CO-saturated KOH was employed in both cell configurations. From the *in situ* Raman spectra, the presence of a surface Cu_2_O peak was observed at OCP in both cell configurations; however, the Cu_2_O peak disappeared upon applying the negative potentials (Fig. 4a, c and S13). These observations are consistent with the discoveries during CO_2_ reduction, indicating that all the *in situ* Raman spectra in CO reduction were also obtained on the metallic Cu surface at negative potentials.

In light of the critical role of *CO in the C–C coupling process, further investigation into the Raman band of *CO was conducted in both cell configurations. When employing the GDE-type spectroelectrochemical flow cell for *in situ* Raman measurements, a weak C–O stretching band centered at ∼2099 cm^−1^ was observed in the high-wavenumber region under an applied potential of −0.2 V (Fig. 4b). As the potential decreased from −0.2 to −0.7 V, we found that the C–O stretching peak in the high-wavenumber region intensified, indicative of an increased CO coverage on the Cu surface. When a more negative potential (<−0.7 V) was applied, the intensity of the C–O stretching peak weakened, which is likely linked to significant consumption of adsorbed CO on the Cu surface caused by the accelerated CO reduction rate. As a comparison, the C–O stretching band was only observed at relatively less negative potentials in the H-type spectroelectrochemical cell (Fig. 4d), but disappeared at more negative potentials, which reflects a low CO coverage on the Cu surface. To obtain a better analysis of the discrepancy in CO coverage between the two cell configurations, the area of the C–O stretching peak at various potentials was quantified by integrating the Gaussian-fitted Raman spectra (Fig. S15). Notably, we found that a substantially higher CO coverage on the Cu surface when using the GDE-type spectroelectrochemical flow cell compared to the H-cell (Fig. S15). These findings show that the difference in mass transport between the GDE-type spectroelectrochemical flow cell and the H-type spectroelectrochemical cell (Fig. 1d) has a significant effect on CO coverage on the catalyst surface when performing CO electrolysis. Additionally, the discrepancy in the line shape of the C–O stretching peak was observed between the GDE-type spectroelectrochemical flow cell (Fig. 4b) and the H-type spectroelectrochemical cell (Fig. 4d), which may be due to distinct CO mass transport that leads to different distributions of CO adsorption sites on the catalyst surface (Fig. S16).

Previous studies suggest that dynamic dipole coupling of CO can introduce a nonlinear relationship between integrated intensity and adsorbate coverage.^36,53^ Thus, to more accurately track changes in CO coverage associated with C_2+_ products, the intensity ratio between the Cu–C stretching and Cu–CO rotation peaks was monitored in prior work.^12,36^ Herein, we also further analyzed the intensity ratio between the Cu–C stretching and Cu–CO rotation peaks (Fig. S17). When employing a GDE-type spectroelectrochemical flow cell (*i.e.* without mass transport limitation) for *in situ* Raman measurements, the application of −0.1 V resulted in the emergence of weak bands at ∼286 and 348 cm^−1^ (Fig. 4a), attributable to the restricted rotation of adsorbed CO and Cu–C stretching of adsorbed CO,^12,45,54^ respectively. As the applied potential decreased from −0.1 to −0.7 V, the integrated area of the Cu–C stretching of adsorbed CO increased progressively, reaching a peak at −0.7 V (Fig. 4a and S18). Notably, we found that the potential-dependent evolution of the intensity ratio between the Cu–C stretching of adsorbed CO and Cu–CO rotation (Fig. S18) closely parallels the variation in the C–O stretching peak (Fig. S15), suggesting that the intensity of the C–O stretching peak may serve as a reliable indicator of CO coverage associated with C_2+_ products on the Cu surface. In contrast, when conducting CO electrolysis in the H-type spectroelectrochemical cell, we did not observe any signals for Cu–C stretching of adsorbed CO and Cu–CO rotation bands across all tested potential ranges (Fig. 4c). This result indicates that CO mass transport limitation in the commonly used H-type spectroelectrochemical cell could significantly influence the detection and analysis of Raman signals related to C–C bond formation. Specifically, CO mass transport limitation results in extremely low CO coverage, which significantly lowers the formation of intermediates that leads to C–C bond formation. Notably, the distinct Raman signals observed in the GDE-type spectroelectrochemical flow cell and the H-type spectroelectrochemical cell are fully consistent with the corresponding differences in electrocatalytic CO reduction performance on Cu surfaces operated in the two different cell configurations (Fig. S19).

Additionally, in the H-type spectroelectrochemical cell, a more pronounced Cu–OH stretching peak was observed compared to that in the GDE-type spectroelectrochemical flow cell (Fig. 4a, c and S20). This may be linked to the fact that only a small amount of CO is adsorbed on the Cu catalyst surface under CO mass transport limitation (H-cell), which results in most of the active sites being occupied by OH species (*i.e.* high OH coverage). This corresponds to a much stronger Cu–OH stretching peak in the H-cell than that in the flow cell.

All the above results in this section manifest that cell configurations could significantly influence the *in situ* Raman detection and analysis of surface-adsorbed intermediates when performing CO reduction. In particular, severe CO mass transport limitation in the commonly used H-type spectroelectrochemical cells substantially reduces the formation and coverage of intermediates related to C_2+_ products, which may significantly affect the detection and analysis of Raman signals during CO reduction and the mechanistic insights for C_2+_ product formation.

## Conclusions

In summary, to uncover the impact of mass transport on the spectral signals and analysis of surface species during electrocatalytic CO_2_/CO reduction, *in situ* Raman spectroscopy was performed using the commonly used H-type spectroelectrochemical cells and GDE-type spectroelectrochemical flow cells, respectively. Our results show that cell configurations have a negligible impact on the *in situ* Raman detection and analysis of surface-adsorbed intermediates during CO_2_ reduction, which indicates that even the commonly used H-type spectroelectrochemical cells can provide reliable *in situ* Raman results for CO_2_ reduction. However, when employing CO reduction, we found that *in situ* Raman signals of surface-adsorbed intermediates in H-cells are significantly distinct from those acquired in GDE-type flow cells (*e.g.*, intermediates related to C_2+_ products). Specifically, the pronounced CO mass transport limitations in commonly used H-type spectroelectrochemical cells markedly suppress the formation and coverage of intermediates associated with C_2+_ products, thereby affecting *in situ* Raman detection results during CO reduction. This work highlights the critical role of mass transport in the *in situ* Raman detection and analysis of surface-adsorbed intermediates, particularly for CO reduction, where mass transport limitations may significantly affect the mechanistic understanding.

## Author contributions

M. M. conceived the original idea of this work. M. M. and J. L. supervised the project. W. Y. synthesized the Cu catalysts, performed SEM and *in situ* Raman experiments. H. B. performed the electrocatalytic tests. M. M., W. Y. and J. L. analyzed the data and wrote the original manuscript. All authors contributed to discussion of the results and manuscript preparation.

## Conflicts of interest

The authors declare no competing financial interests.

## Supplementary Material

SC-017-D5SC07260C-s001

## Data Availability

All data needed to evaluate the conclusions in the paper are present in the paper and/or the supplementary information (SI). Additional data related to this paper may be requested from the authors. Supplementary information is available. See DOI: https://doi.org/10.1039/d5sc07260c.

## References

[cit1] Seh Z. W., Kibsgaard J., Dickens C. F., Chorkendorff I. B., Norskov J. K., Jaramillo T. F. (2017). Combining theory and experiment in electrocatalysis: Insights into materials design. Science.

[cit2] Yan T. X., Chen X. Y., Kumari L., Lin J. L., Li M. L., Fan Q., Chi H. Y., Meyer T. J., Zhang S., Ma X. B. (2023). Multiscale CO_2_ Electrocatalysis to C_2+_ Products: Reaction Mechanisms, Catalyst Design, and Device Fabrication. Chem. Rev..

[cit3] Ma M., Seger B. (2024). Rational Design of Local Reaction Environment for Electrocatalytic Conversion of CO_2_ into Multicarbon Products. Angew. Chem., Int. Ed..

[cit4] Birdja Y. Y., Pérez-Gallent E., Figueiredo M. C., Göttle A. J., Calle-Vallejo F., Koper M. T. M. (2019). Advances and challenges in understanding the electrocatalytic conversion of carbon dioxide to fuels. Nat. Energy.

[cit5] Nitopi S., Bertheussen E., Scott S. B., Liu X., Engstfeld A. K., Horch S., Seger B., Stephens I. E. L., Chan K., Hahn C., Norskov J. K., Jaramillo T. F., Chorkendorff I. (2019). Progress and Perspectives of Electrochemical CO_2_ Reduction on Copper in Aqueous Electrolyte. Chem. Rev..

[cit6] Kuhl K. P., Cave E. R., Abram D. N., Jaramillo T. F. (2012). New insights into the electrochemical reduction of carbon dioxide on metallic copper surfaces. Energy Environ. Sci..

[cit7] Jouny M., Luc W., Jiao F. (2018). General Techno-Economic Analysis of CO_2_ Electrolysis Systems. Ind. Eng. Chem. Res..

[cit8] Jhong H. R., Ma S. C., Kenis P. J. A. (2013). Electrochemical conversion of CO_2_ to useful chemicals: current status, remaining challenges, and future opportunities. Curr. Opin. Chem. Eng..

[cit9] Gunathunge C. M., Ovalle V. J., Li Y. W., Janik M. J., Waegele M. M. (2018). Existence of an Electrochemically Inert CO Population on Cu Electrodes in Alkaline pH. ACS Catal..

[cit10] An H. Y., Wu L. F., Mandemaker L. D. B., Yang S., de Ruiter J., Wijten J. H. J., Janssens J. C. L., Hartman T., van der Stam W., Weckhuysen B. M. (2021). Sub-Second Time-Resolved Surface-Enhanced Raman Spectroscopy Reveals Dynamic CO Intermediates during Electrochemical CO_2_ Reduction on Copper. Angew. Chem., Int. Ed..

[cit11] Zhao Z.-H., Ren D. (2024). Unravelling the Effect of Crystal Facet of Derived-Copper Catalysts on the Electroreduction of Carbon Dioxide under Unified Mass Transport Condition. Angew. Chem., Int. Ed..

[cit12] Zhan C., Dattila F., Rettenmaier C., Bergmann A., Kühl S., García-Muelas R., López N., Cuenya B. R. (2021). Revealing the CO Coverage-Driven C-C Coupling Mechanism for Electrochemical CO_2_ Reduction on Cu_2_O Nanocubes via Operando Raman Spectroscopy. ACS Catal..

[cit13] Gao J., Zhang H., Guo X. Y., Luo J. S., Zakeeruddin S. M., Ren D., Gratzel M. (2019). Selective C-C Coupling in Carbon Dioxide Electroreduction via Efficient Spillover of Intermediates As Supported by Operando Raman Spectroscopy. J. Am. Chem. Soc..

[cit14] Ma M., Deng W., Xu A., Hochfilzer D., Qiao Y., Chan K., Chorkendorff I., Seger B. (2022). Local reaction environment for selective electroreduction of carbon monoxide. Energy Environ.
Sci..

[cit15] Luc W., Fu X. B., Shi J. J., Lv J. J., Jouny M., Ko B. H., Xu Y. B., Tu Q., Hu X. B., Wu J. S., Yue Q., Liu Y. Y., Jiao F., Kang Y. J. (2019). Two-dimensional copper nanosheets for electrochemical reduction of carbon monoxide to acetate. Nat. Catal..

[cit16] Zhu Y. P., Wang J. L., Chu H., Chu Y. C., Chen H. M. (2020). In Situ Operando Studies for Designing Next-Generation Electrocatalysts. ACS Energy Lett..

[cit17] Iijima G., Inomata T., Yamaguchi H., Ito M., Masuda H. (2019). Role of a Hydroxide Layer on Cu Electrodes in Electrochemical CO_2_ Reduction. ACS Catal..

[cit18] Zhu S., Jiang B., Cai W. B., Shao M. (2017). Direct Observation on Reaction Intermediates and the Role of Bicarbonate Anions in CO_2_ Electrochemical Reduction Reaction on Cu Surfaces. J. Am. Chem. Soc..

[cit19] Sheng H., Oh M. H., Osowiecki W. T., Kim W., Alivisatos A. P., Frei H. (2018). Carbon Dioxide Dimer Radical Anion as Surface Intermediate of Photoinduced CO_2_ Reduction at Aqueous Cu and CdSe Nanoparticle Catalysts by Rapid-Scan FT-IR Spectroscopy. J. Am. Chem. Soc..

[cit20] Gunathunge C. M., Li X., Li J. Y., Hicks R. P., Ovalle V. J., Waegele M. M. (2017). Spectroscopic Observation of Reversible Surface Reconstruction of Copper Electrodes under CO_2_ Reduction. J. Phys. Chem. C.

[cit21] Heidary N., Ly K. H., Kornienko N. (2019). Probing CO_2_ Conversion Chemistry on Nanostructured Surfaces with Operando Vibrational Spectroscopy. Nano Lett..

[cit22] Kas R., Ayemoba O., Firet N. J., Middelkoop J., Smith W. A., Cuesta A. (2019). In-Situ Infrared Spectroscopy Applied to the Study of the Electrocatalytic Reduction of CO_2_: Theory, Practice and Challenges. ChemPhysChem.

[cit23] Bodappa N., Su M., Zhao Y., Le J. B., Yang W. M., Radjenovic P., Dong J. C., Cheng J., Tian Z. Q., Li J. F. (2019). Early Stages of Electrochemical Oxidation of Cu(111) and Polycrystalline Cu Surfaces Revealed by in Situ Raman Spectroscopy. J. Am. Chem. Soc..

[cit24] Zhao Y., Chang X., Malkani A. S., Yang X., Thompson L., Jiao F., Xu B. (2020). Speciation of Cu Surfaces During the Electrochemical CO Reduction Reaction. J. Am. Chem. Soc..

[cit25] Dunwell M., Lu Q., Heyes J. M., Rosen J., Chen J. G., Yan Y., Jiao F., Xu B. (2017). The Central Role of Bicarbonate in the Electrochemical Reduction of Carbon Dioxide on Gold. J. Am. Chem. Soc..

[cit26] Dong J. C., Zhang X. G., Briega-Martos V., Jin X., Yang J., Chen S., Yang Z. L., Wu D. Y., Feliu J. M., Williams C. T., Tian Z. Q., Li J. F. (2019). In situ Raman spectroscopic evidence for oxygen reduction reaction intermediates at platinum single-crystal surfaces. Nat. Energy.

[cit27] Chang X., Malkani A., Yang X., Xu B. (2020). Mechanistic Insights into Electroreductive C-C Coupling between CO and Acetaldehyde into Multicarbon Products. J. Am. Chem. Soc..

[cit28] Firet N. J., Smith W. A. (2017). Probing the Reaction Mechanism of CO_2_ Electroreduction over Ag Films via Operando Infrared Spectroscopy. ACS Catal..

[cit29] Zhang D. A., Liu X., Zhao Y., Zhang H., Rudnev A. V., Li J. F. (2025). In situ Raman spectroscopic studies of CO_2_ reduction reactions: from catalyst surface structures to reaction mechanisms. Chem. Sci..

[cit30] Pérez-Gallent E., Figueiredo M. C., Calle-Vallejo F., Koper M. T. M. (2017). Spectroscopic Observation of a Hydrogenated CODimer Intermediate During CO Reduction on Cu(100) Electrodes. Angew. Chem., Int. Ed..

[cit31] Chang X. X., Vijay S., Zhao Y. R., Oliveira N. J., Chan K. R., Xu B. J. (2022). Understanding the complementarities of surface-enhanced infrared and Raman spectroscopies in CO adsorption and electrochemical reduction. Nat. Commun..

[cit32] Zhao Y., Zhang X. G., Bodappa N., Yang W. M., Liang Q., Radjenovica P. M., Wang Y. H., Zhang Y. J., Dong J. C., Tian Z. Q., Li J. F. (2022). Elucidating electrochemical CO_2_ reduction reaction processes on Cu(hkl) single-crystal surfaces by in situ Raman spectroscopy. Energy Environ. Sci..

[cit33] Chernyshova I. V., Somasundaran P., Ponnurangam S. (2018). On the origin of the elusive first intermediate of CO_2_ electroreduction. Proc. Natl. Acad. Sci. U. S. A..

[cit34] Burdyny T., Smith W. A. (2019). CO_2_ reduction on gas-diffusion electrodes and why catalytic performance must be assessed at commercially-relevant conditions. Energy Environ. Sci..

[cit35] Ma M., Clark E. L., Therkildsen K. T., Dalsgaard S., Chorkendorff I., Seger B. (2020). Insights into the carbon balance for CO_2_ electroreduction on Cu using gas diffusion electrode reactor designs. Energy Environ. Sci..

[cit36] Yan W., Wu T. T., Liu J., Zheng Z., Ma M. (2025). Mass Transport-Dependent C-C Bond Formation for CO Electroreduction with Alkali Cations. J. Am. Chem. Soc..

[cit37] Chang X. X., Zhao Y. R., Xu B. J. (2020). pH Dependence of Cu Surface Speciation in the Electrochemical CO Reduction Reaction. ACS Catal..

[cit38] Zang J. Y., Ye W. T., Liu Q. L., Meng J. H., Yang W. X. (2025). Plasmonic-Promoted Formation of Surface Adsorbed Stochastic CO during Electrochemical CO_2_ and CO Reduction on Cu at Extreme Low Overpotentials. J. Am. Chem. Soc..

[cit39] Li J., Chang X. X., Zhang H. C., Malkani A. S., Cheng M. J., Xu B. J., Lu Q. (2021). Electrokinetic and in situ spectroscopic investigations of CO electrochemical reduction on copper. Nat. Commun..

[cit40] Clark E. L., Resasco J., Landers A., Lin J., Chung L. T., Walton A., Hahn C., Jaramillo T. F., Bell A. T. (2018). Standards and Protocols for Data Acquisition and Reporting for Studies of the Electrochemical Reduction of Carbon Dioxide. ACS Catal..

[cit41] Weng L. C., Bell A. T., Weber A. Z. (2018). Modeling gas-diffusion electrodes for CO_2_ reduction. Phys. Chem. Chem. Phys..

[cit42] Ma M., Kim S., Chorkendorff I., Seger B. (2020). Role of ion-selective membranes in the carbon balance for CO_2_ electroreduction via gas diffusion electrode reactor designs. Chem. Sci..

[cit43] Zheng Z., Yao Y. F., Yan W., Bu H. Y., Huang J. F., Ma M. (2024). Mechanistic Insights into the Abrupt Change of Electrolyte in CO_2_ Electroreduction. ACS Catal..

[cit44] Scott S. B., Hogg T. V., Landers A. T., Maagaard T., Bertheussen E., Lin J. C., Davis R. C., Beeman J. W., Higgins D., Drisdell W. S., Hahn C., Mehta A., Seger B., Jaramillo T. F., Chorkendorff I. (2019). Absence of Oxidized Phases in Cu under CO Reduction Conditions. ACS Energy Lett..

[cit45] An H. Y., de Ruiter J., Wu L. F., Yang S., Meirer F., van der Stam W., Weckhuysen B. M. (2023). Spatiotemporal Mapping of Local Heterogeneities during Electrochemical Carbon Dioxide Reduction. JACS Au.

[cit46] de Ruiter J., Benning V. R. M., Yang S., den Hartigh B. J., Wang H., Prins P. T., Dorresteijn J. M., Janssens J. C. L., Manna G., Petukhov A. V., Weckhuysen B. M., Rabouw F. T., van der Stam W. (2025). Multiscale X-ray scattering elucidates activation and deactivation of oxide-derived copper electrocatalysts for CO_2_ reduction. Nat. Commun..

[cit47] Moradzaman M., Mul G. (2021). In Situ Raman Study of Potential-Dependent Surface Adsorbed Carbonate, CO, OH, and C Species on Cu Electrodes During Electrochemical Reduction of CO_2_. ChemElectroChem.

[cit48] Jouny M., Luc W., Jiao F. (2018). High-rate electroreduction of carbon monoxide to multi-carbon products. Nat. Catal..

[cit49] Gunathunge C. M., Ovalle V. J., Waegele M. M. (2017). Probing promoting effects of alkali cations on the reduction of CO at the aqueous electrolyte/copper interface. Phys. Chem. Chem. Phys..

[cit50] Cao Y. F., Chen Z., Li P. H., Ozden A., Ou P. F., Ni W. Y., Abed J., Shirzadi E., Zhang J. Q., Sinton D., Ge J., Sargent E. H. (2023). Surface hydroxide promotes CO_2_ electrolysis to ethylene in acidic conditions. Nat. Commun..

[cit51] Wiebe R., Gaddy V. L. (1940). The solubility of carbon dioxide in water at various temperatures from 12 to 40° and at pressures to 500 atmospheres: Critical phenomena. J. Am. Chem. Soc..

[cit52] Shao F., Wong J. K., Low Q. H., Iannuzzi M., Li J. G., Lan J. G. (2022). In situ spectroelectrochemical probing of CO redox landscape on copper single-crystal surfaces. Proc. Natl. Acad. Sci. U. S. A..

[cit53] Borguet E., Dai H. L. (1994). Site-specific properties and dynamical dipole coupling of CO molecules adsorbed on a vicinal Cu(100) surface. J. Chem. Phys..

[cit54] Ren D., Gao J., Zakeeruddin S. M., Grätzel M. (2021). New Insights into the Interface of Electrochemical Flow Cells for Carbon Dioxide Reduction to Ethylene. J. Phys. Chem. Lett..

