# Supplementary material for: Mass transport-dependent in situ Raman detection in CO/CO2 electrolysis
Source: Chem Sci. 2025 Dec 16;17(7):3563–71. doi: 10.1039/d5sc07260c (PMC12724533; doi:10.1039/d5sc07260c)
Supplement: SC-017-D5SC07260C-s001 [file SC-017-D5SC07260C-s001.pdf]

Supplementary Information (SI) for Chemical Science.
This journal is © The Royal Society of Chemistry 2025

Supplementary Information

Mass Transport-Dependent *In Situ* Raman Detection in CO/CO_2 Electrolysis

Wen Yan,[1] Hangyu Bu,[1] Xinjuan Du,[1] Beining Xu,[1] Jia Liu,[2*] and Ming Ma[1,*]

[1]School of Chemical Engineering and Technology, Xi'an Jiaotong University, Xi'an 710049, People's Republic of China

[2]Instrument Analysis Center, Xi'an Jiaotong University, Xi'an 710049, People's Republic of China

*Author to whom correspondence should be addressed.

E-mail address: liujia6j@xjtu.edu.cn and mingma@xjtu.edu.cn

Supplementary Methods

Materials

Potassium hydroxide (KOH, 99.99% metals basis), Potassium bicarbonate ($KHCO_3$, ≥99.99% metals basis, 99.7-100.5% dry basis) were purchased from Aladdin. All chemicals were used in this work without further purification. Anion exchange membrane (AEM, Fumasep FAA-3-PK-75) and gas-diffusion electrode (GDE, Sigracet 39 BB) were purchased from Fuel Cell Store. The electrolyte solutions were prepared with 18.2 MΩ cm ultrapure (UP) water obtained from Hitech Instruments CO., Ltd (Master-S15UV).

Electrode Preparation and Characterization

Cu catalysts were deposited onto the microporous layers of gas diffusion electrodes (GDEs) via direct current magnetron sputtering at 25 W, using a high-purity Cu target (99.9999%) under an argon pressure of 0.5 Pa (Supplementary Fig. 1). The thickness of the Cu layer was controlled by adjusting the sputtering deposition time. In this study, all Cu layers were prepared with a fixed deposition time of 20 min to ensure identical catalyst thickness on the GDEs. To determine the deposition rate and thickness of the Cu layer, deposition was also conducted on polished silicon substrates for 20 min. A cross-sectional image of the Cu layer on silicon obtained using field emission scanning electron microscopy (FE-SEM, MAIA3 LMH, TESCAN) exhibits that a Cu layer with approximately 200 nm was observed on the silicon substrate after 20 min of deposition (as shown in Supplementary Fig. 2), corresponding to a deposition rate of 10 nm min^{-1}. In addition, the optical reflectance of the pristine gas-diffusion electrode (GDE) and the Cu-deposited GDE was measured using a UV–Vis spectrophotometer (PerkinElmer Lambda 950) equipped with a 150 mm integrating sphere (Supplementary Fig. 3). Reflectance spectra were collected over the wavelength range of 200–1000 nm.

Electrochemical Measurements

The electrochemical measurements were performed using a Biologic SP-150e electrochemical station. All experiments were conducted at room temperature and ambient pressure. Firstly, CO/CO_2 electrolysis was carried out in a custom-made spectroelectrochemical flow cell, comprising three chambers: a gas chamber, a catholyte chamber, and an anolyte chamber. For the flow cell setup, the Cu catalysts deposited on microporous carbon layers of GDEs were used as the working electrode (cathode) and a

graphite electrode as the counter electrode (anode). An anion exchange membrane (AEM, Fumasep FAA-3-PK-75) was employed to separate the catholyte and anolyte chambers. The flow cell assembly consisted of the Cu cathode, AEM and graphite anode, and the liquid electrolytes (30 ml catholyte and 30 ml anolyte) were circulated in both catholyte and anolyte chambers at a constant flowrate (14 ml min^{-1}) using two peristaltic pumps (Kamoer). CO gas was continuously supplied to the gas chamber at a constant flowrate (20 ml min^{-1}) and passed behind the GDE using a mass flow controller. In the three-electrode system, a Ag/AgCl or Hg/HgO reference electrode was inserted into the catholyte chamber. Additionally, CO/CO_2 electrolysis was performed in a conventional spectroelectrochemical H-cell with two chambers: the catholyte and anolyte chambers. In the H-cell setup, the Cu catalysts deposited on microporous carbon layers of GDEs were used as the working electrode (cathode), with graphite as the counter electrode (anode). The AEM separated the catholyte and anolyte chambers, and the electrolytes (30 ml catholyte and 30 ml anolyte) were circulated in both catholyte and anolyte chambers at a constant flowrate of 14 ml min^{-1} using two peristaltic pumps. Prior to the experiments, CO or CO_2 was purged into catholyte and anolyte for 1 h at a flowrate of 20 ml min^{-1} to ensure the complete saturation of the electrolytes. Before *in situ* Raman measurements, linear sweep voltammetry (LSV) was used to investigate the CO_2/CO reduction on Cu catalysts using two *in situ* Raman configurations with distinct mass transport conditions at -0.1 to -0.9 V vs RHE, scan rate: 0.05 V/s^{-1} (Supplementary Figs. 6 and 12).

Gas Product Analysis

Gas products formed during CO reduction (COR) were analyzed using a gas chromatograph (GC) equipped with a thermal conductivity detector (TCD) and a flame ionization detector (FID). Argon served as the carrier gas throughout the measurements. The TCD was employed to quantify H_2, CH_4, and C_2H_4, while the FID coupled with a methanizer enabled sensitive detection of hydrocarbon products even at trace concentrations.

During electrolysis, the gas outlet was periodically directed to the GC sampling loop for quantitative analysis at 11 min intervals. Each electrolysis experiment was conducted for 1 h, with gas-phase analyses performed at 10, 21, 32, 43 and 54 min. The Faradaic efficiency for each gaseous product was evaluated using the equation below:

$$FE_{gas}(\%) = \frac{Q_{gas}}{Q_{total}} \times 100\% = \frac{n \cdot C_{gas} \cdot \emptyset \cdot \frac{P}{RT} \cdot F}{I} \times 100\%$$

(1)

where Q_{gas} and Q_{total} represent the charge associated with the formation of a specific gaseous product and the total charge passed through the working electrode, respectively. Here, n denotes the number of electrons required to generate one molecule of the corresponding gas product. C_{gas} is the gas concentration determined by gas chromatography, Φ is the flow rate of the outlet gas, P is the ambient pressure, R is the ideal gas constant, T is the absolute temperature, F is the Faraday constant, and I is the applied current.

Liquid Product Analysis

After completion of the CO reduction experiments, the catholyte and anolyte were collected and analyzed using high-performance liquid chromatography (HPLC, Agilent 1260) equipped with a refractive index detector (RID) and an Aminex HPX-87H column (Bio-Rad Laboratories). A 5 mM aqueous H_2SO_4 solution was employed as the eluent. For each run, 50 µL of the liquid sample was injected via an autosampler, and the column temperature was maintained at 55 °C. The separated liquid products were identified and quantified by the RID. The Faradaic efficiency for each liquid product was evaluated using the equation below:

$$FE_{liquid}(\%) = \frac{Q_{liquid}}{Q_{total}} \times 100\% = \frac{n \cdot C_{liquid} \cdot V \cdot F}{I \cdot t} \times 100\%$$

(2)

where Q_{liquid} and Q_{total} denote the charges associated with liquid product formation and the total charge passed through the working electrode, respectively. n represents the number of electrons required to generate one molecule of the corresponding liquid product. C_{liquid} is the concentration of the liquid product determined by HPLC. V refers to the actual measured volumes of the catholyte and anolyte after high-rate CO electrolysis. F is the Faraday constant, I is the applied current, and t is the electrolysis duration.

In situ Raman Measurements

A comparative study of the electrocatalytic CO_2/CO reduction on the Cu catalysts was conducted using two distinct types of Raman spectroelectrochemical cells, with *in situ* Raman spectroscopy monitoring (Renishaw Invia Qontor). *In situ* Raman experiments were performed in a conventional spectroelectrochemical H-cell and a GDE-type spectroelectrochemical flow cell, respectively. Supplementary Fig. 4a and b present the detailed components and the fully assembled configuration of conventional spectroelectrochemical H-cell. Additionally, the detailed components and the assembled configuration of GDE-type spectroelectrochemical flow cell are shown in Supplementary Fig. 4c and d, respectively. After combining with Raman setup, our full *in situ* Raman experimental setup under different mass-transport conditions can be found in Supplementary Fig. 5. For all *in situ* Raman experiments, a cathodic GDE coated with a Cu catalyst layer of uniform thickness (~ 200 nm) was used as the working electrode to ensure reliable comparison of surface-adsorbed species signals. The excitation source was a 785 nm laser with a power of 6.75 mW and a grating of 1200 grooves/mm, coupled with a 50×L objective (Leica). Each Raman spectrum was acquired with an acquisition time of 1 s and 10 times of accumulation. Standard silicon wafers are used for calibration before each test.

In this work, the background correction of Raman spectra was performed using the "Subtract baseline" function in the WiRE 5.3 software, which is dedicated data acquisition and processing software developed by Renishaw for its Raman spectrometers. Specifically, the *Fitting mode* was set to *Intelligent fitting*, in which the algorithm automatically excludes peak regions and fits the baseline only to non-peak points. The *Baseline type* was set to *Intelligent polynomial*, with a *Polynomial order* of 11 and a *Noise tolerance* of 2.0, to effectively remove slowly varying background signals while preserving true Raman features. After fitting, the calculated baseline was subtracted from each spectrum to obtain the final background-corrected data. During data processing, we visually inspected baseline-corrected spectra across the dataset, which ensures that the subtraction does introduce systematic artifacts (such as persistent negative troughs) or distort true peaks. Any spectra showing suspicious features were reprocessed with slightly adjusted noise-tolerance or polynomial-order settings and rechecked. The parameter set described above represents the final values that were applied uniformly to all spectra presented in the manuscript.

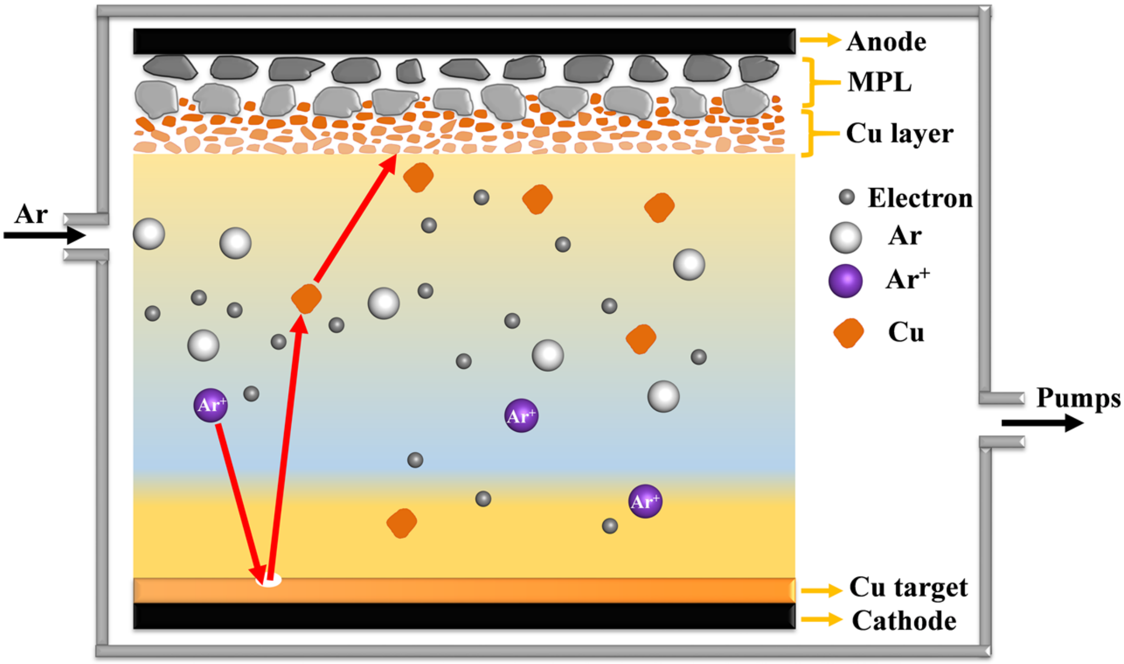

Supplementary Figure 1. Schematic of Cu deposition via magnetron sputtering with a Cu target.

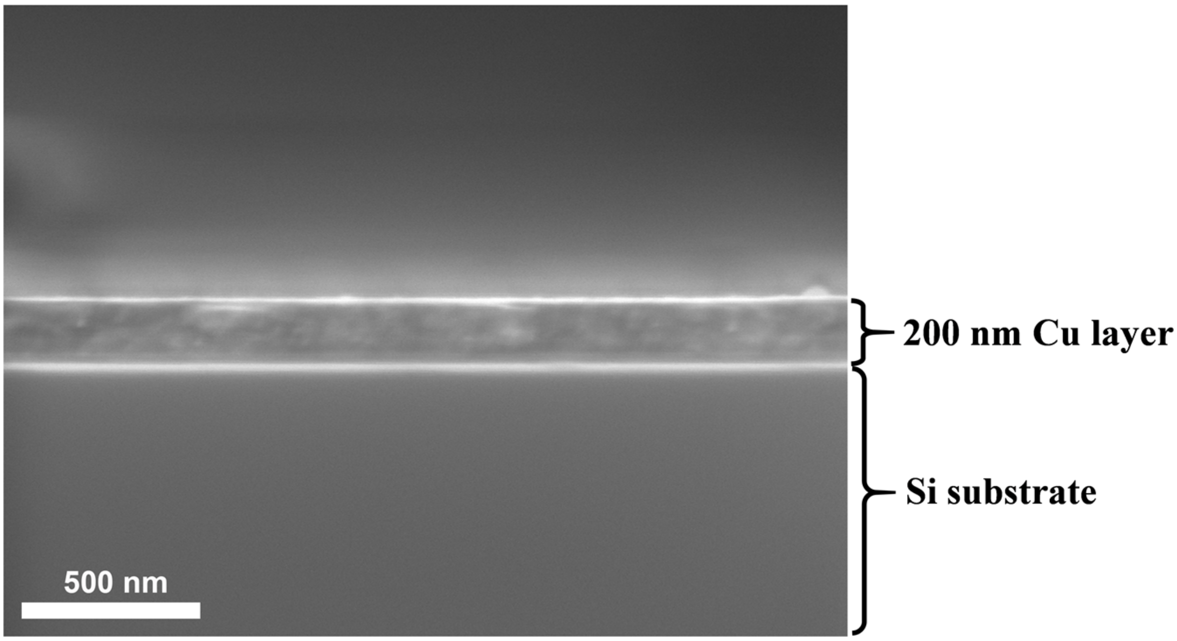

Supplementary Figure 2. Cross-sectional SEM image of Cu layer deposited on Si by magnetron sputtering for 20 min.

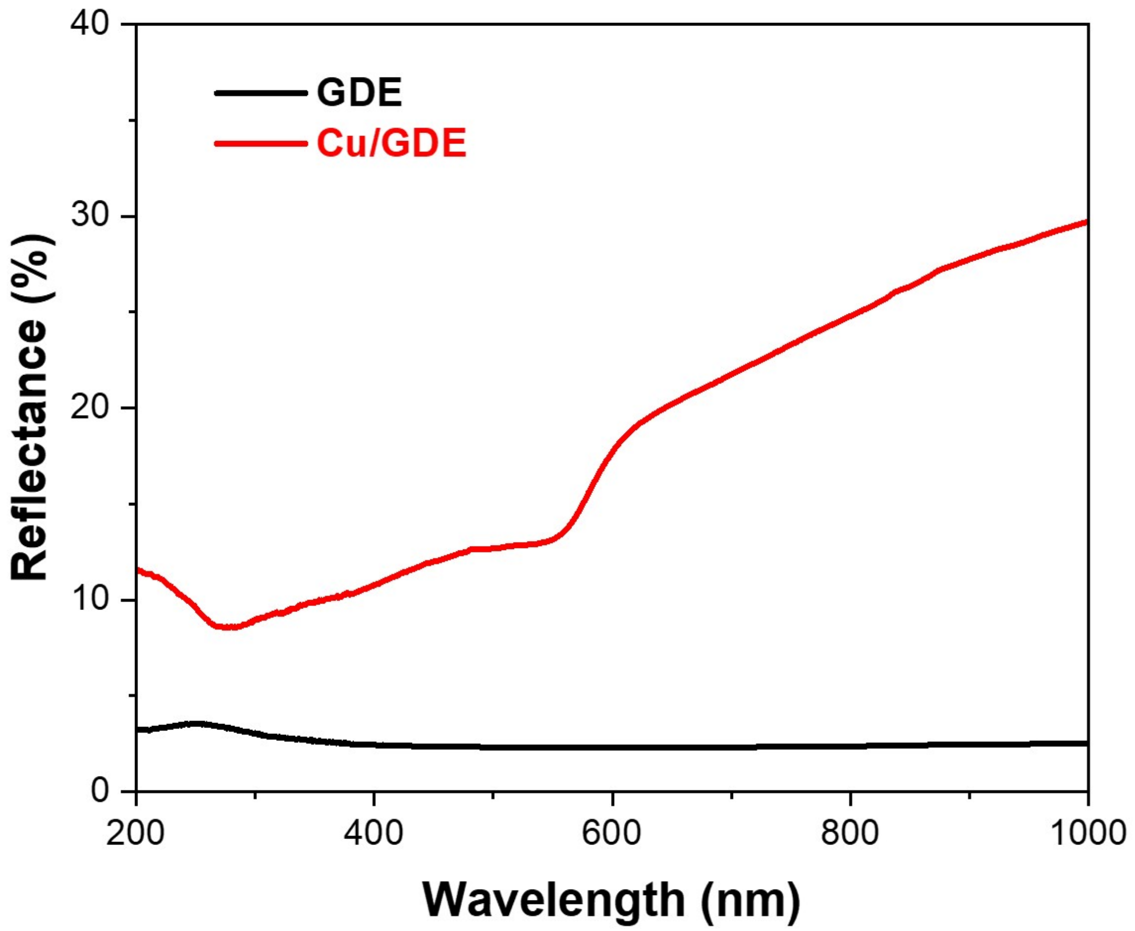

Supplementary Figure 3. UV–Vis reflectance spectra of the pristine gas-diffusion electrode (GDE) and the Cu-deposited GDE.

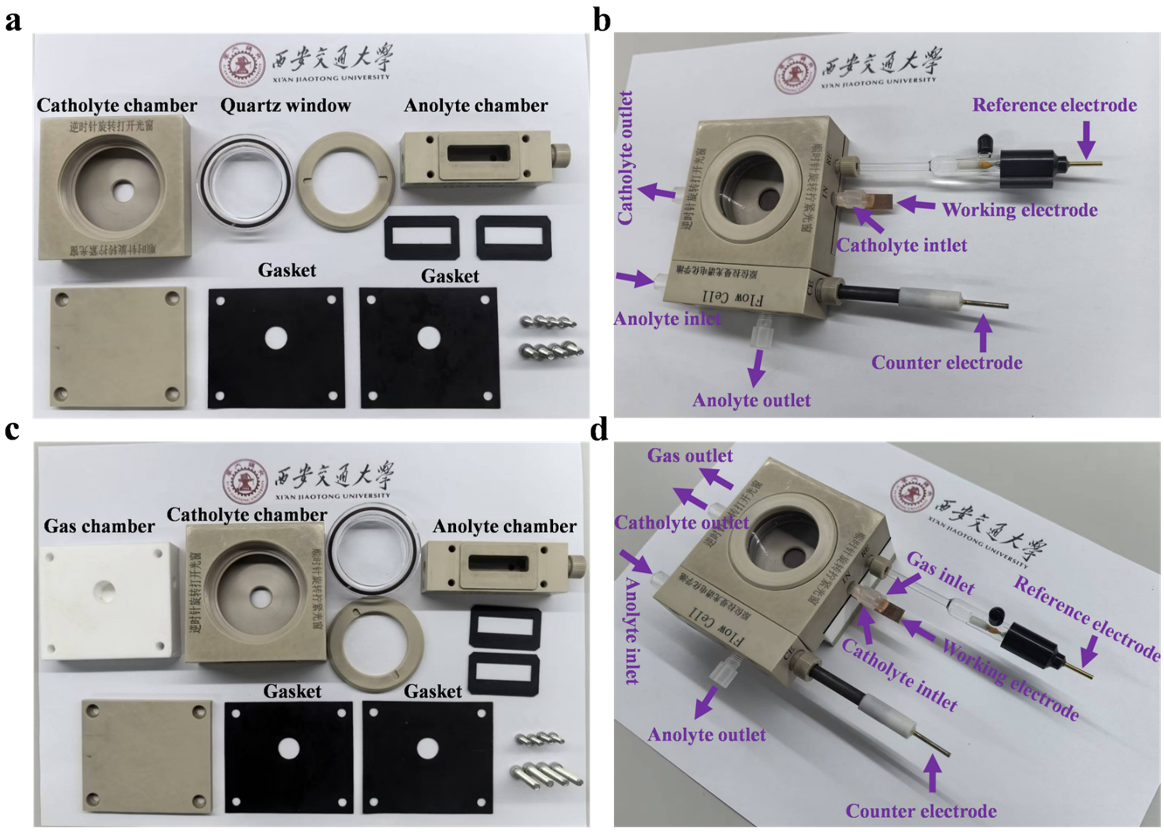

Supplementary Figure 4. Photographs of the spectroelectrochemical cells used for *in situ* Raman measurements. (a, b) Components and assembled configuration of the H-type cell. (c, d) Components and assembled configuration of the GDE-type flow cell.

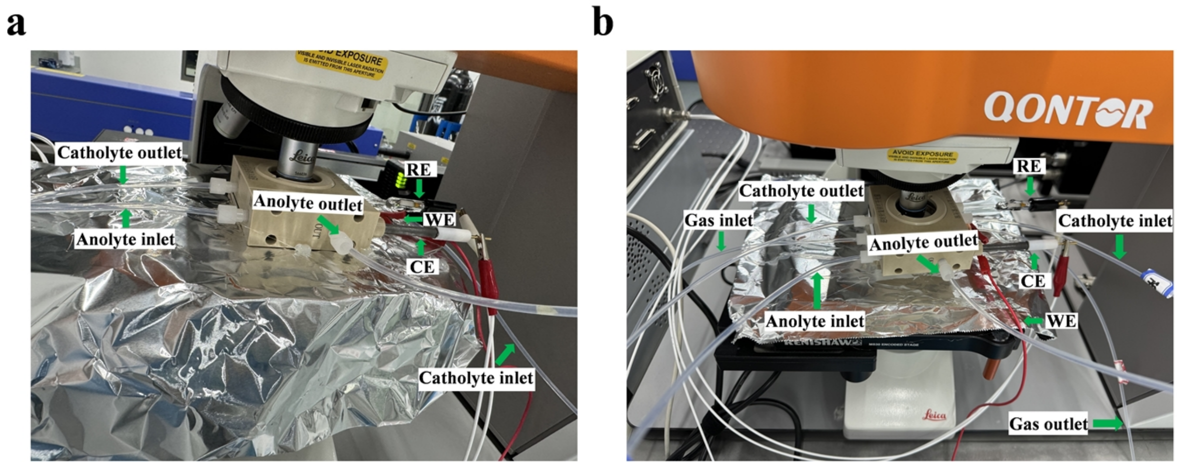

Supplementary Figure 5. *In situ* Raman experimental setup. Photographs of the assembled *in situ* spectroelectrochemical cells: (a) H-cell and (b) flow cell.

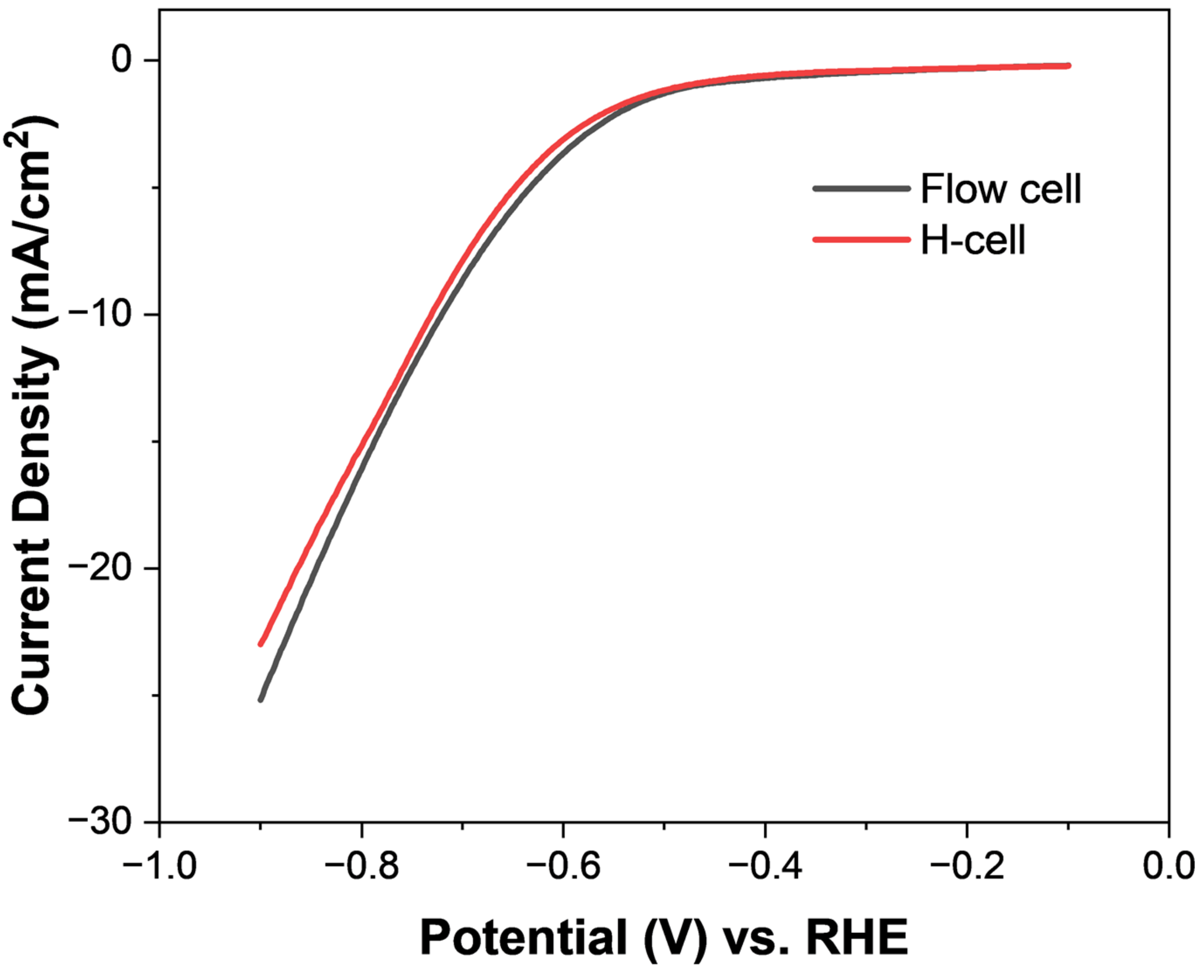

Supplementary Figure 6. Linear sweep voltammetry (LSV) of electrochemical CO_2 reduction on Cu catalysts in 0.5 M $KHCO_3$ solution using two *in situ* Raman configurations with distinct mass transport conditions at -0.1 to -0.9 V (vs RHE, no *iR* correction), scan rate: 0.05 V/s^{-1} .

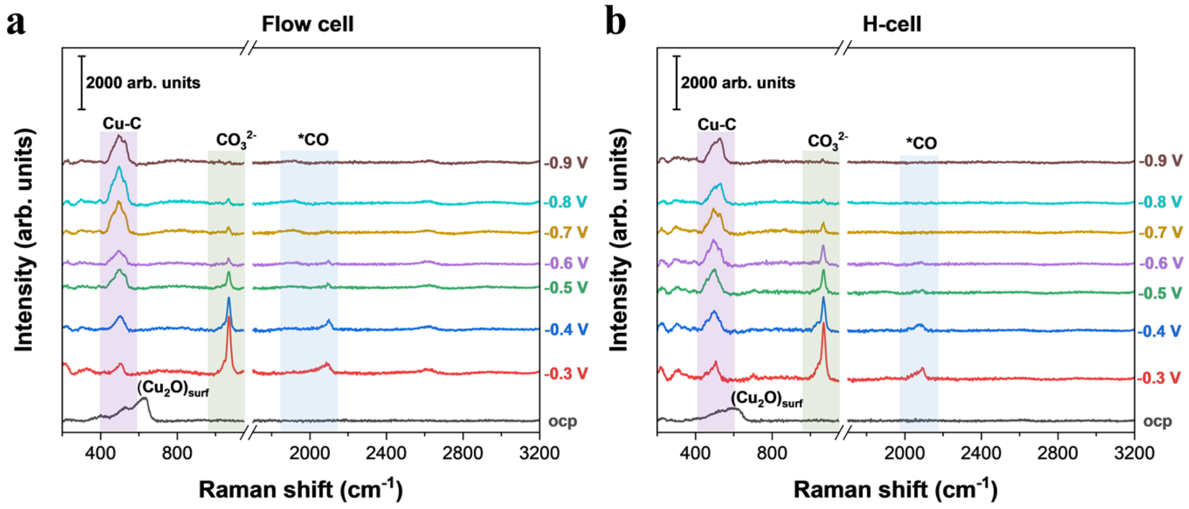

Supplementary Figure 7. *In situ* Raman spectroscopy of electrochemical CO_2 reduction on Cu catalysts using two *in situ* Raman configurations with distinct mass transport conditions. (a) Raman spectra on Cu catalysts at OCP and -0.3 ~ -0.9 V (vs RHE, no *iR* correction) in 0.5 M $KHCO_3$ collected in the GDE-type spectroelectrochemical flow cell. (b) Raman spectra on Cu catalysts at OCP and -0.3 ~ -0.9 V (vs RHE, no *iR* correction) in 0.5 M $KHCO_3$ collected in the H-type spectroelectrochemical cell.

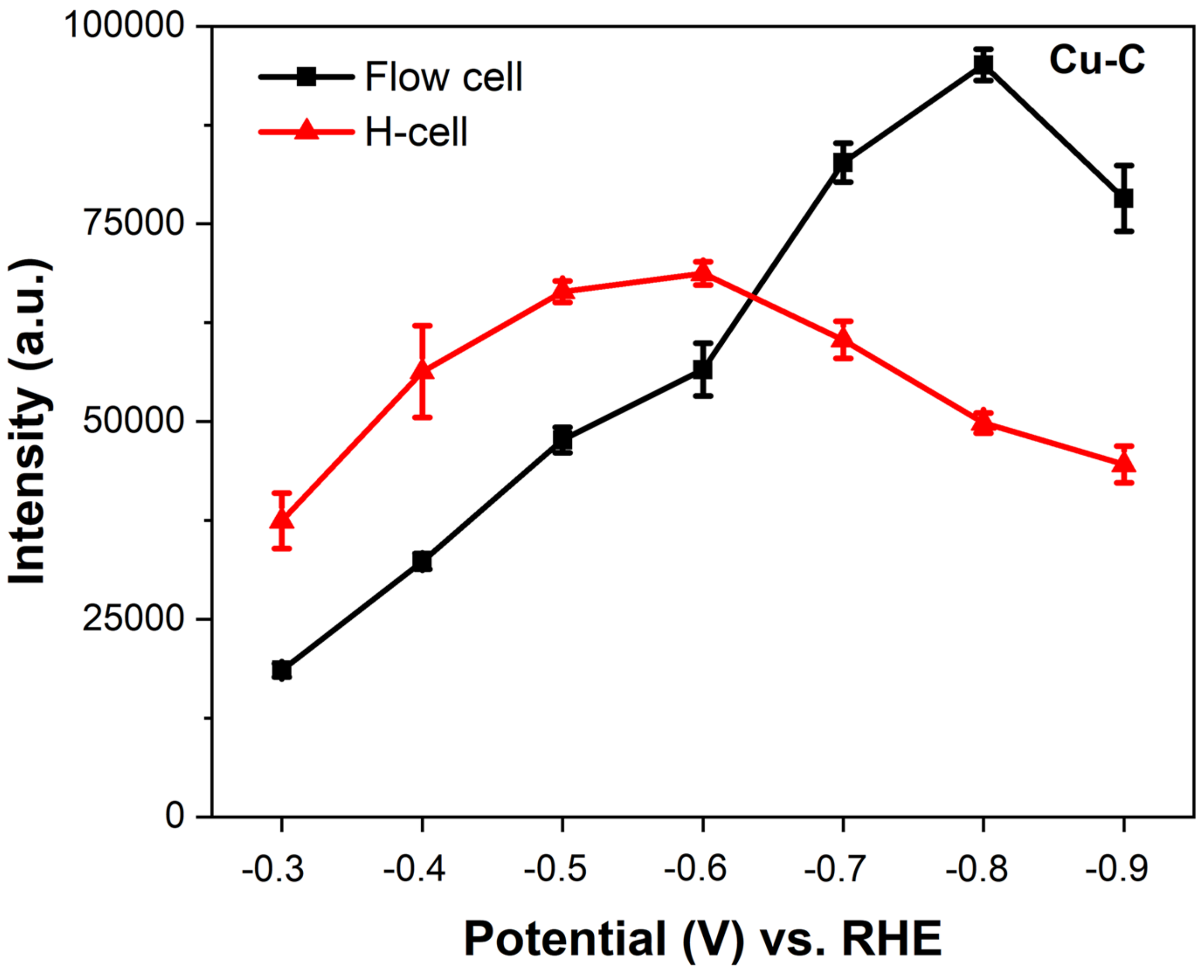

Supplementary Figure 8. Comparison of the intensity of Cu-C band on Cu catalysts in CO_2-saturated 0.5 M $KHCO_3$ collected in the GDE-type spectroelectrochemical flow cell and the H-type spectroelectrochemical cell at various potentials. The error bars represent the standard deviation from at least three independent measurements.

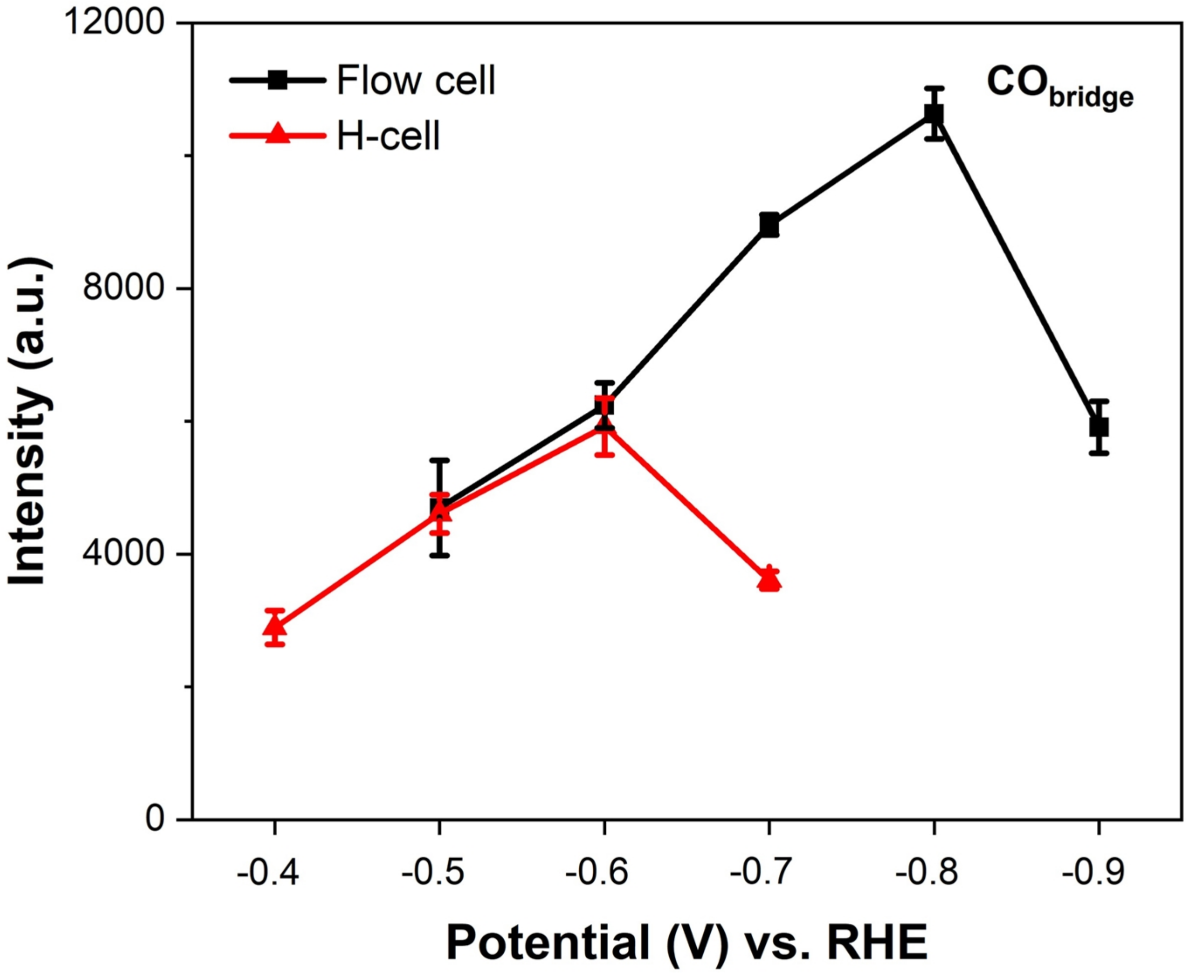

Supplementary Figure 9. Comparison of the intensity of CO_{bridge} band on Cu catalysts in CO_2-saturated 0.5 M $KHCO_3$ collected in the GDE-type spectroelectrochemical flow cell and the H-type spectroelectrochemical cell at various potentials. The error bars represent the standard deviation from at least three independent measurements.

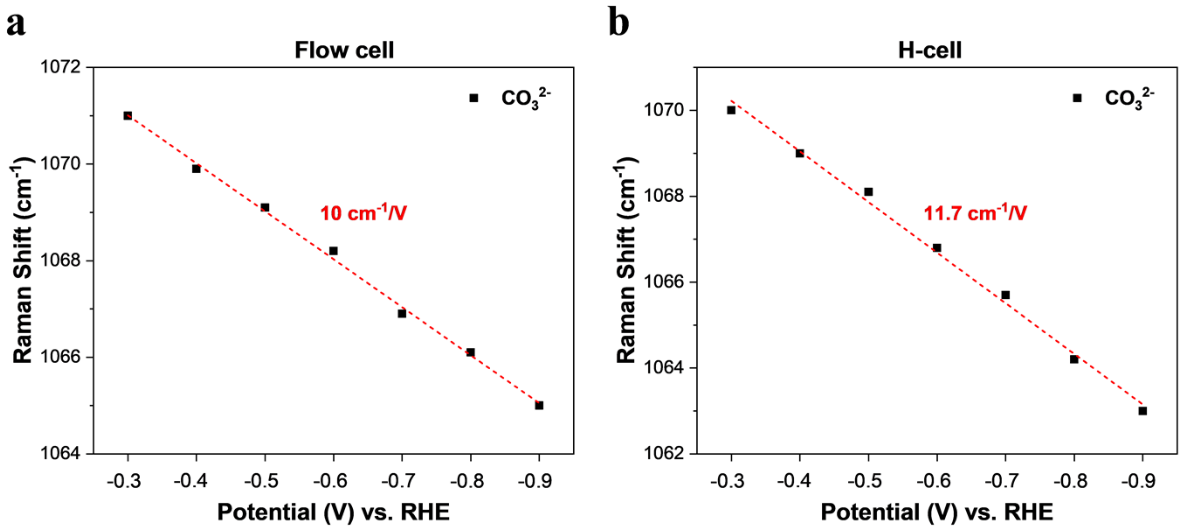

Supplementary Figure 10. Effect of electrode potential on the peak frequency of adsorbed carbonate on Cu catalysts in CO_2-saturated 0.5 M $KHCO_3$. (a) The carbonate peak frequency in Raman spectra as a function of electrode potential collected in the GDE-type spectroelectrochemical flow cell. (b) The carbonate peak frequency in Raman spectra as a function of electrode potential collected in the H-type spectroelectrochemical cell. The carbonate band redshifts 11.7 cm^{-1}/V in the H-type spectroelectrochemical cell, in line with prior work,[1, 2] confirming that the Stark effect originates from chemisorbed carbonate species on the Cu surface.

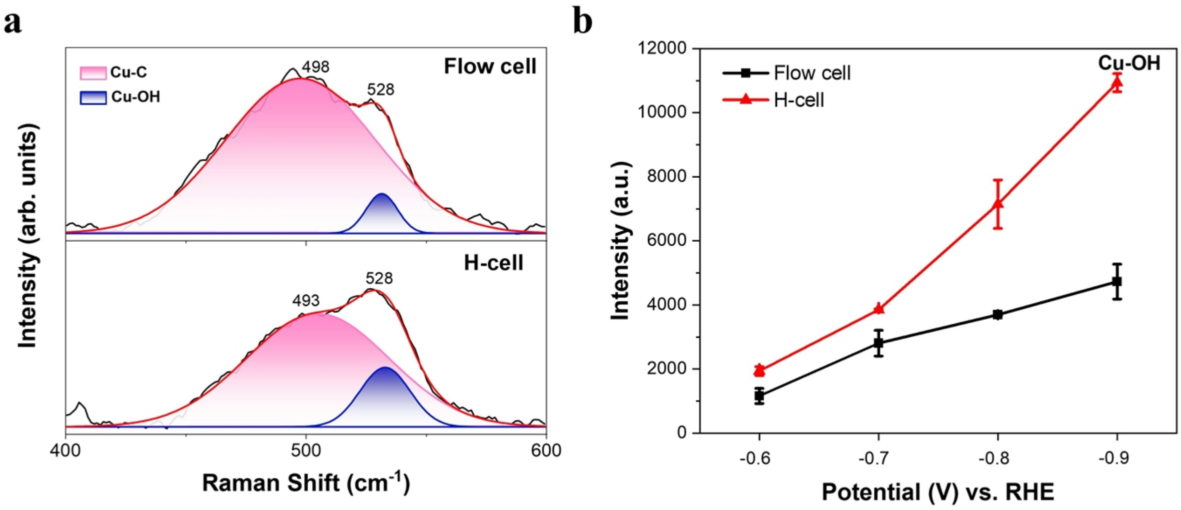

Supplementary Figure 11. *In situ* Raman spectra of electrochemical CO_2 reduction on Cu catalysts collected in the GDE-type spectroelectrochemical flow cell and the H-type spectroelectrochemical cell. (a) Deconvoluted spectra of the low-wavenumber region of Figure 2a and 2d at an applied of potential -0.9 V. (b) Comparison of the intensity of Cu-OH band on Cu catalysts in CO_2-saturated 0.5 M $KHCO_3$ collected in the GDE-type spectroelectrochemical flow cell and the H-type spectroelectrochemical cell at various potentials. The error bars represent the standard deviation from at least three independent measurements.

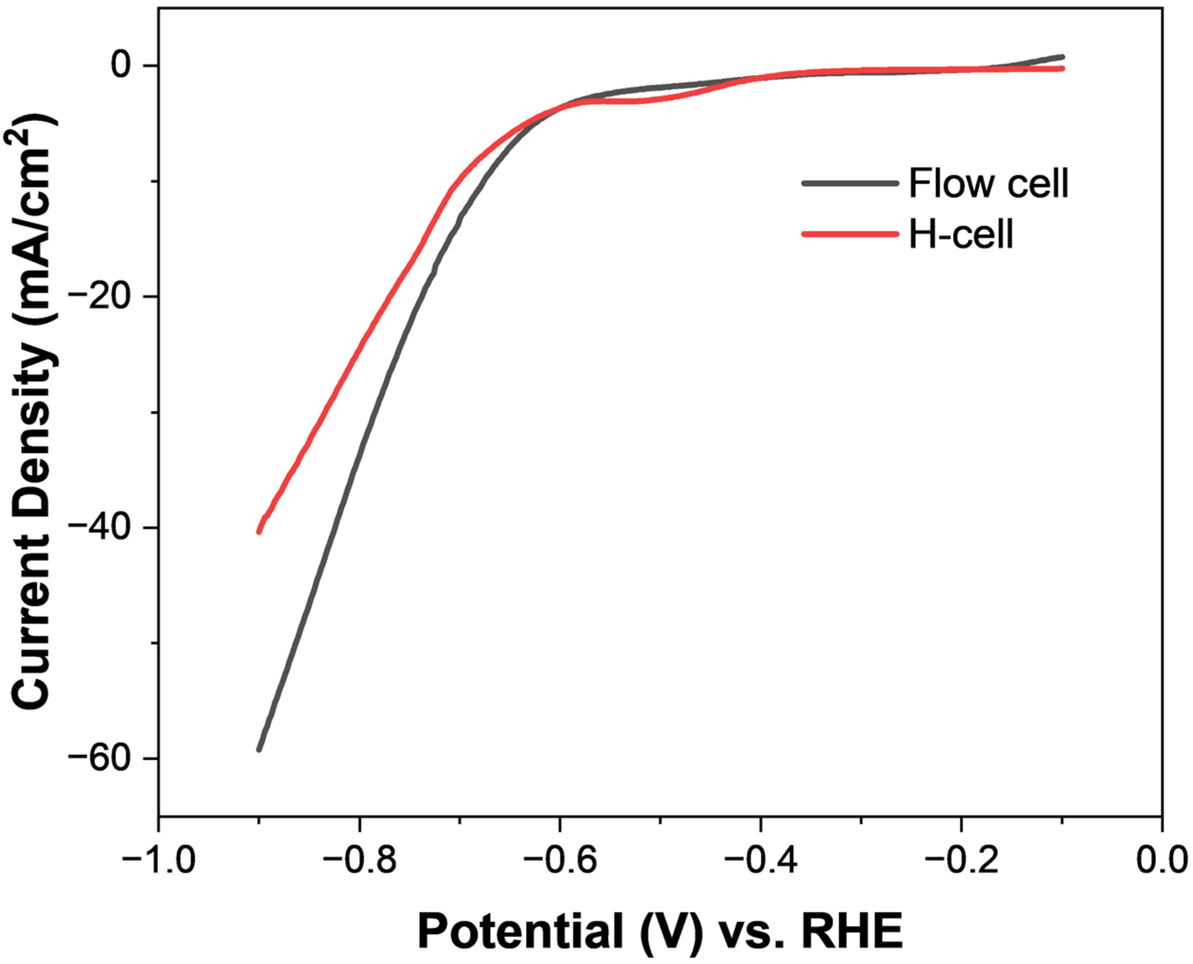

Supplementary Figure 12. Linear sweep voltammetry (LSV) of electrochemical CO reduction on Cu catalysts in 0.5 M KOH solution using two *in situ* Raman configurations with distinct mass transport conditions at -0.1 to -0.9 V (vs RHE, no *iR* correction), scan rate: 0.05 V/s^{-1} .

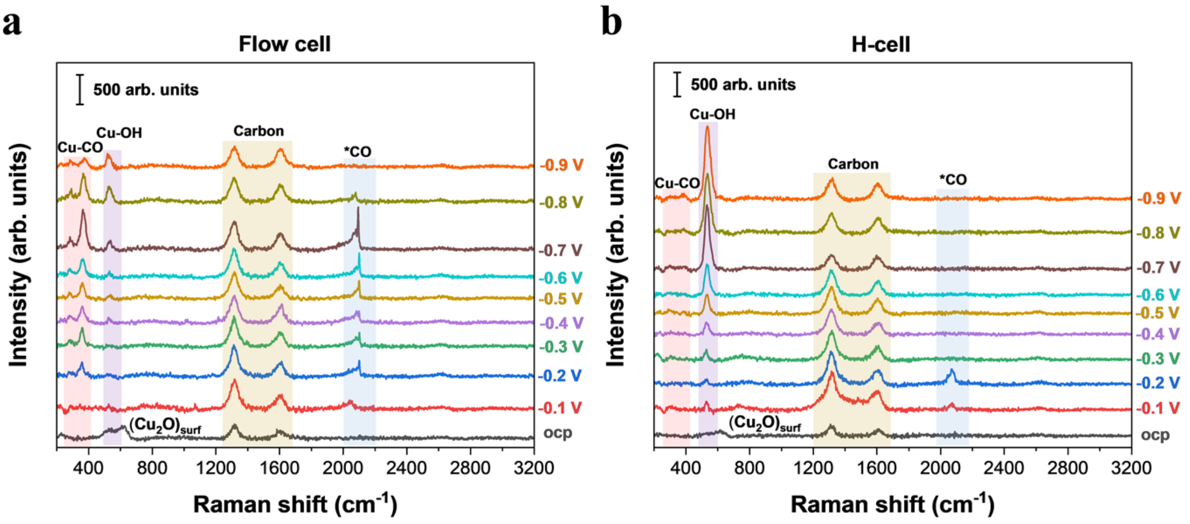

Supplementary Figure 13. *In situ* Raman spectroscopy of electrochemical CO reduction on Cu catalysts using two *in situ* Raman configurations with distinct mass transport conditions. (a) Raman spectra on Cu catalysts at OCP and -0.1 ~ -0.9 V (vs RHE, no *iR* correction) in 0.5 M KOH collected in the GDE-type spectroelectrochemical flow cell. (b) Raman spectra on Cu catalysts at OCP and -0.1 ~ -0.9 V (vs RHE, no *iR* correction) in 0.5 M KOH collected in the H-type spectroelectrochemical cell.

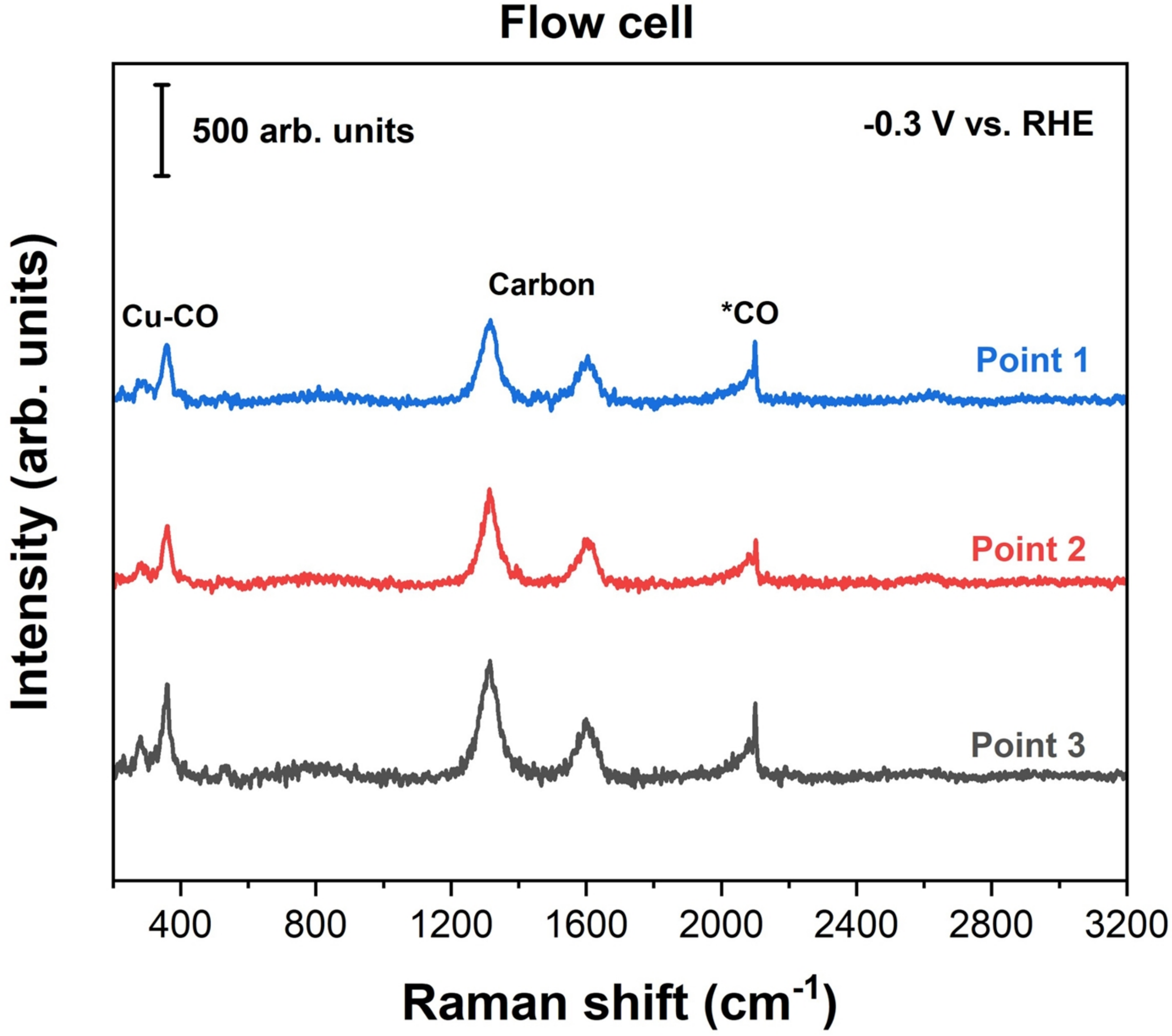

Supplementary Figure 14. *In situ* Raman spectroscopy of electrochemical CO reduction on Cu catalysts in 0.5 M KOH at -0.3 V vs RHE collected in the GDE-type spectroelectrochemical flow cell. Three spectra at three random spots on the sample were collected at each potential.

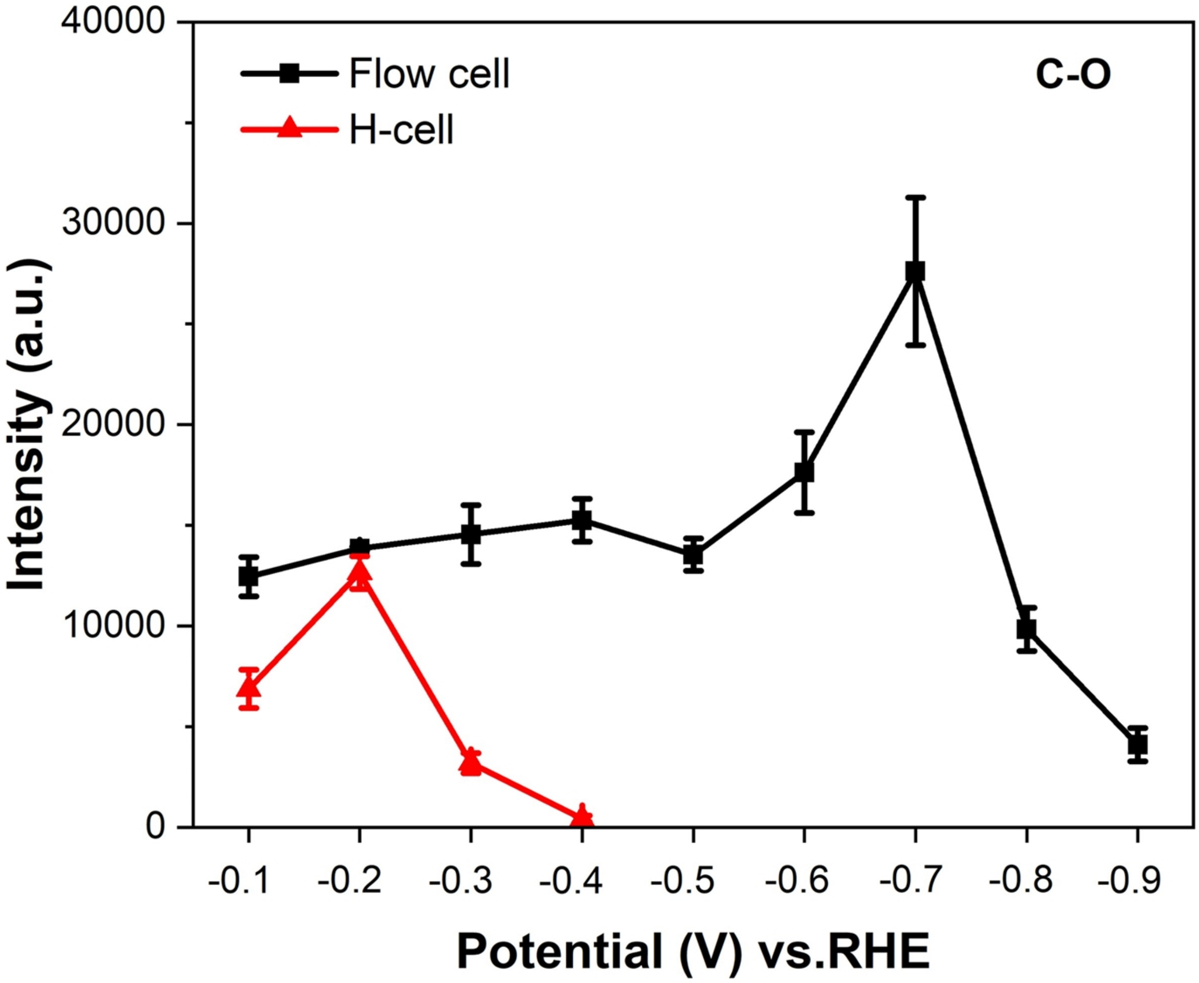

Supplementary Figure 15. Comparison of the intensity of C-O band on Cu catalysts in CO-saturated 0.5 M KOH collected in the H-type spectroelectrochemical cell and the GDE-type spectroelectrochemical flow cell at various potentials. The error bars represent the standard deviation from at least three independent measurements.

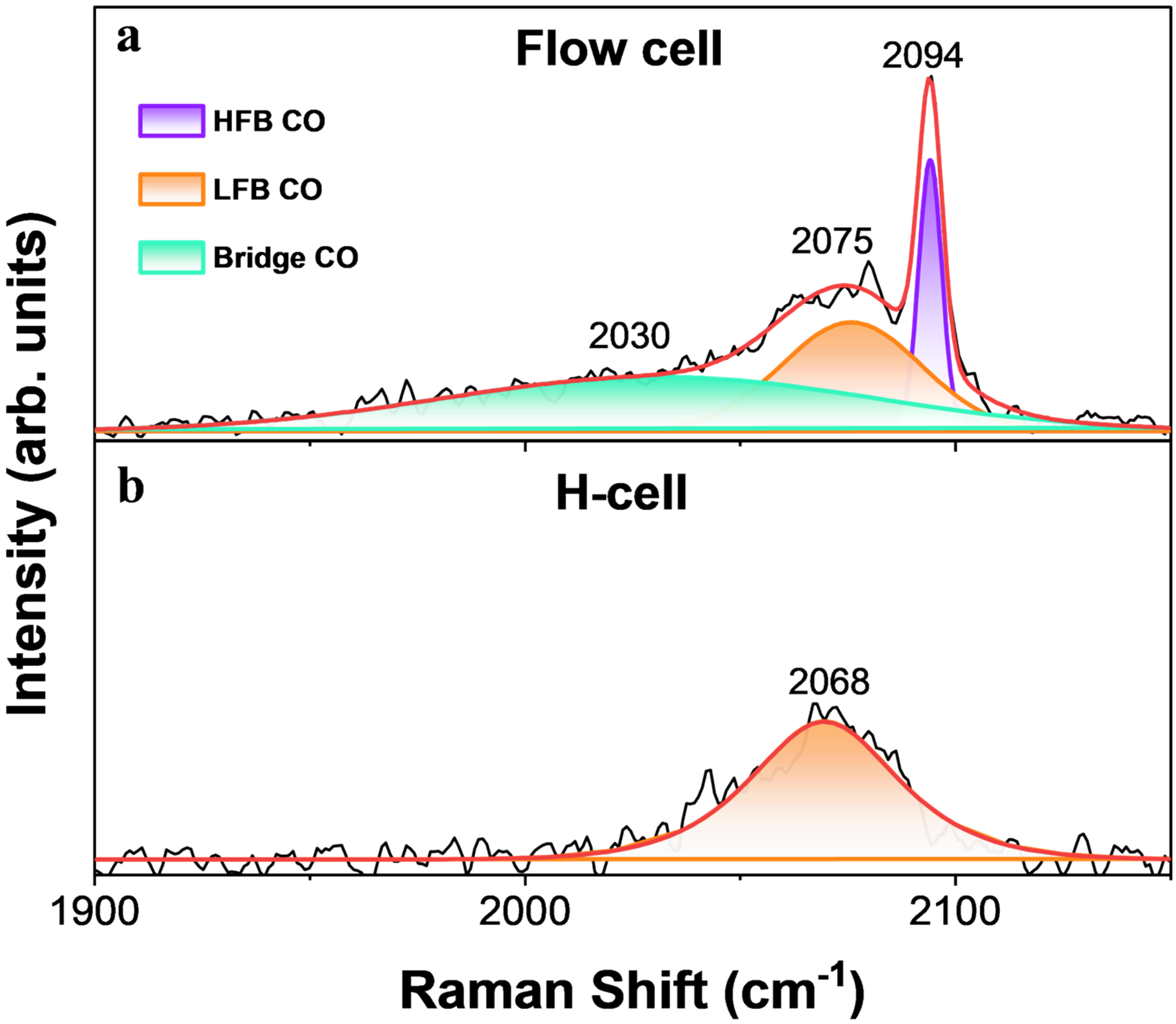

Supplementary Figure 16. *In situ* Raman spectra of electrochemical CO reduction on Cu catalysts collected in the GDE-type spectroelectrochemical flow cell and the H-type cell. (a) Deconvoluted *in situ* Raman spectra of the C–O stretching region (Flow cell), obtained from Figure 4b using Gaussian peak fitting at an applied potential of –0.7 V vs RHE. (b) Deconvoluted *in situ* Raman spectra of the C–O stretching region (H-cell), obtained from Figure 4d using Gaussian peak fitting at an applied potential of –0.2 V vs RHE.

The C–O stretching vibrational peak is highly sensitive to the local coordination environment of adsorbed CO,[3] including specific adsorption sites (e.g., terrace/defect sites and neighboring adsorbed CO). Therefore, differences in line shape of C-O stretching peak between Figures 4b and 4d suggest distinct CO adsorption environments on the catalyst surface under the two cell configurations.

To further elucidate this point, we performed Gaussian deconvolution of the C–O stretching region for both spectra (Supplementary Figure 16). For the flow cell configuration, the C–O stretching band can be deconvoluted into three peaks: (1) bridge-bonded CO (CO_{bridge}) at ~2030 cm^{-1}, (2) a low-frequency band linear CO (LFB-CO) at ~2075 cm^{-1}, and (3) a high-frequency band linear CO (HFB-CO) at ~2094 cm^{-1}. In contrast, the spectra collected in the H-type cell can only be deconvoluted into a single peak, corresponding to LFB-CO centered at ~2068 cm^{-1}. According to previous studies,[4,5] the LFB-CO at ~2075 cm^{-1} is attributed to CO_{atop} on terrace sites, whereas the HFB-CO at ~2094 cm^{-1} is associated with CO_{atop} on isolated defect sites. The broad line shape of the C-O stretching peak in the flow cell configuration indicates the presence of CO on a wider distribution of surface sites, including terrace sites, defect sites, and neighboring adsorbed CO, which is consistent with the high CO availability caused by accelerated CO mass transport via GDEs in the flow cell. In contrast, in the H-cell, the CO mass-transport limitation results in significantly lower CO surface coverage. Under such conditions, CO tends to occupy only the most energetically favorable terrace atop sites, resulting in a single, narrower C–O stretching band. Thereby, the distinct Raman line shapes observed in Figures 4b and 4d should be correlated with the discrepancy in CO mass transport in the two different cell configurations, which gives rise to different distributions of CO adsorption sites on the Cu catalyst surface.

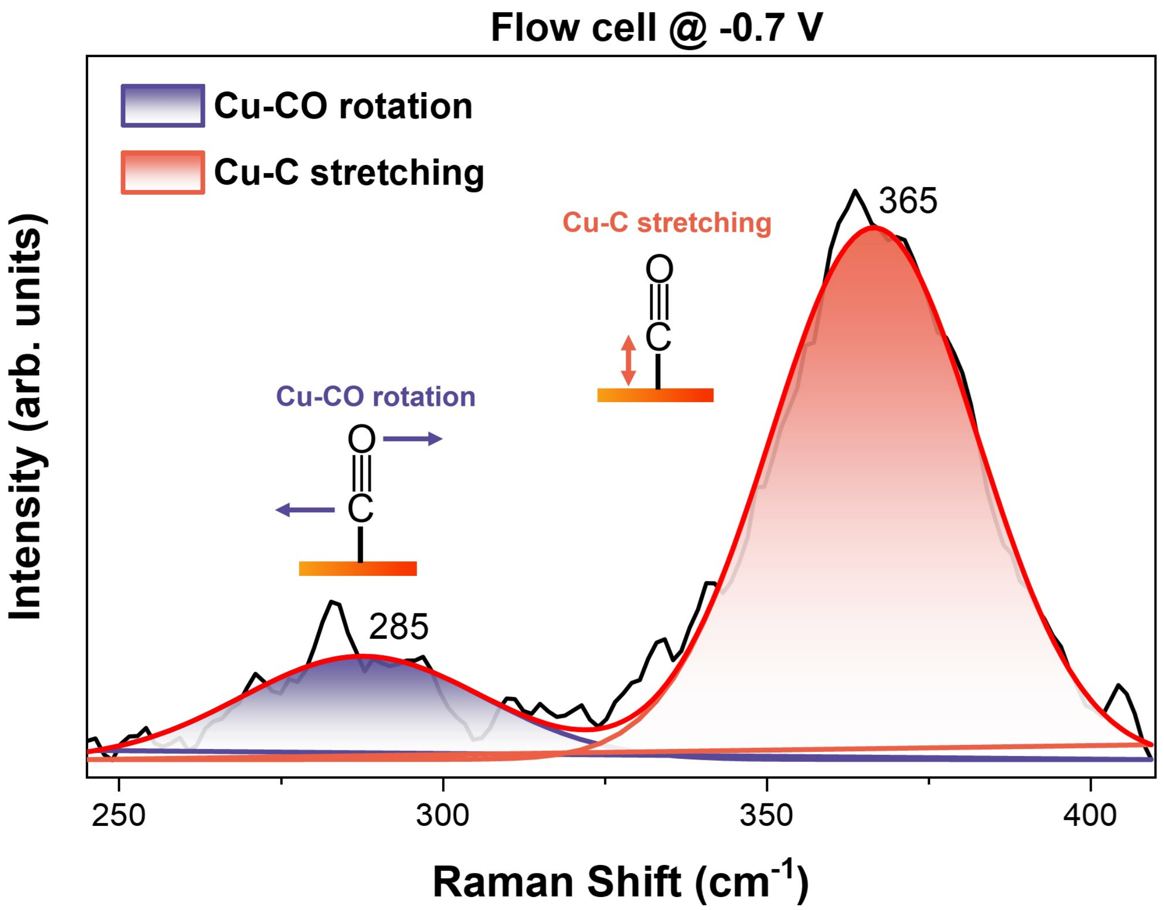

Supplementary Figure 17. *In situ* Raman spectra of electrochemical CO reduction on Cu catalysts collected in the GDE-type spectroelectrochemical flow cell. Deconvoluted *in situ* Raman spectra of the Cu–CO region from Figure 4a, obtained by Gaussian peak fitting at an applied potential of –0.7 V vs RHE. The peaks centered at 285 and 365 cm^{-1} have been attributed to the restricted rotation of adsorbed CO and Cu–C stretching of adsorbed CO.[5-7]

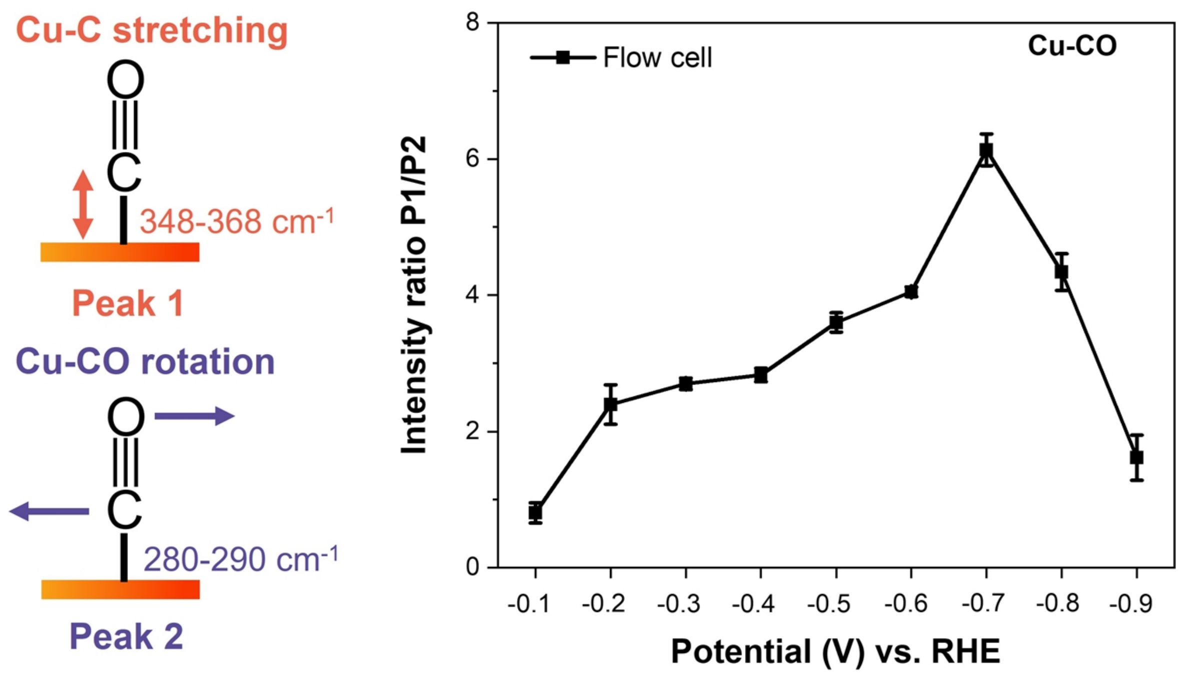

Supplementary Figure 18. Probing surface CO coverage–driven CO–CO coupling. (a) Schematic illustration of two distinct CO adsorption configurations on the Cu surface. (b) Potential-dependent intensity ratio of Cu–C stretching of adsorbed CO (P1) to the restricted rotation of adsorbed CO (P2), extracted from the *in situ* Raman spectra in Figure 4a. The error bars represent the standard deviation from at least three independent measurements.

A comparison of electrocatalytic CO reduction performance in the H-cell and flow cell configurations

To directly compare the CO electroreduction performance in the two cell configurations, we evaluated the catalytic performance in both the H-cell and the GDE-type flow cell. As shown in Supplementary Fig. 19, the H-cell consistently exhibits much lower selectivity and activity toward multi-carbon (C_{2+}) products than those in the GDE-type flow cell at the same applied potentials. Since C_{2+} formation is closely linked to the CO surface coverage, the suppressed C_{2+} performance in the H-cell reflects insufficient CO availability on the catalyst surface. In contrast, the GDE-type flow cell configuration provides a continuous and rapid CO supply, maintaining a higher surface CO coverage and enabling markedly enhanced C_{2+} selectivity and activity. Thereby, the significant difference in the C_{2+} selectivity and activity under same potential between the H-cell and GDE-type flow cell align well with the distinct Raman signals observed in the two cell designs (*e.g.* significant discrepancy in CO coverage).

Furthermore, the kinetic trends further support the distinct mass-transport environments. In the H-cell configuration, the total current density for C_{2+} products increased as the potential switched from −1.2 V to −1.25 V vs SHE, and then gradually decreased at more negative potentials than -1.25 V vs SHE (Supplementary Fig. 19b). This turnover behavior is indicative of CO mass-transport limitations. By comparison, in the GDE-type flow cell configuration, the total current density for C_{2+} products enhanced monotonically from −1.2 V to −1.55 V vs SHE (Supplementary Fig. 19d), demonstrating that no CO mass-transport limitations are encountered.

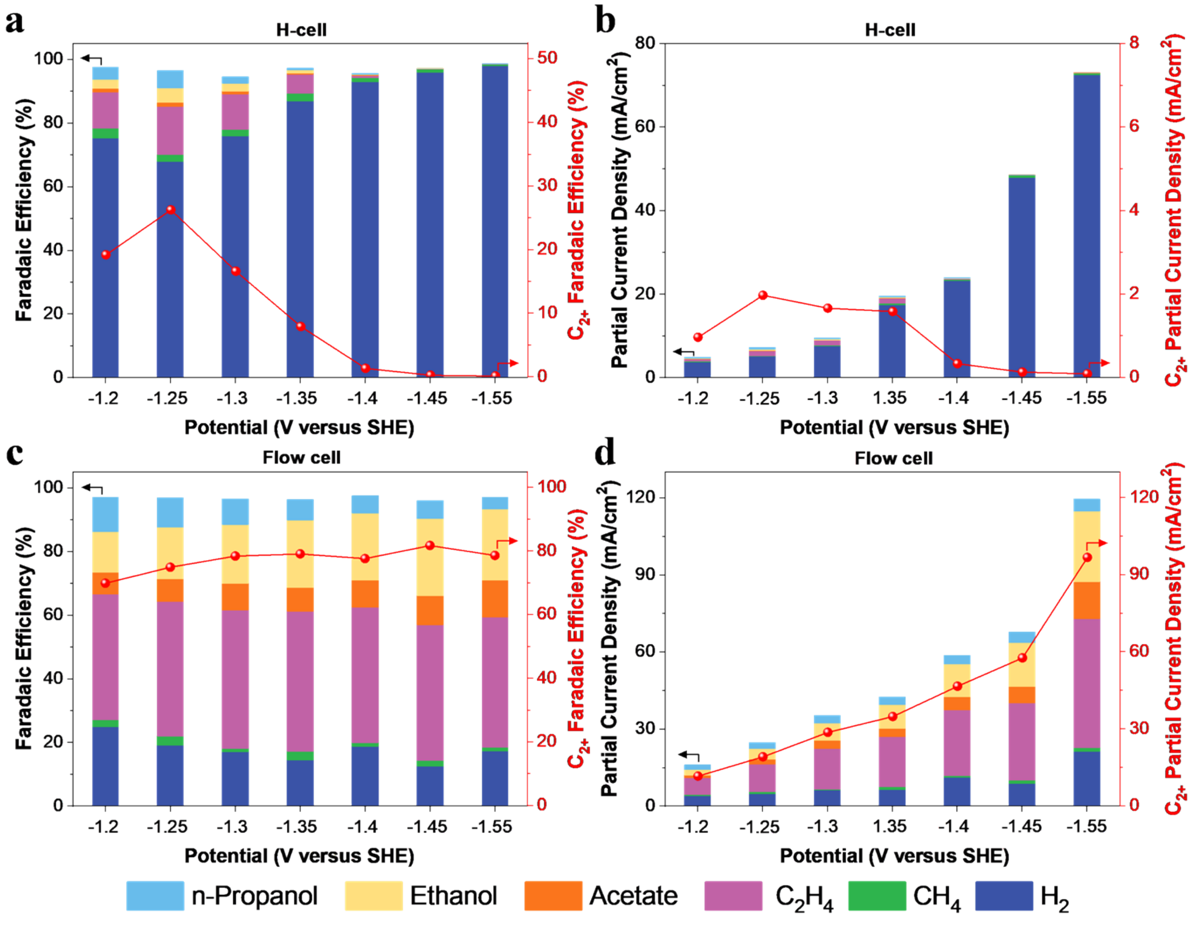

Supplementary Figure 19. The electrocatalytic performance of CO reduction on Cu catalysts in 0.5 M KOH using two commonly used electrochemical configurations. Faradaic efffciencies of major products as a function of applied potentials in (a) the H-cell, and (c) the GDE-type flow cell. Corresponding partial current densities of major products as a function of applied potentials in (b) the H-cell, and (d) the flow cell.

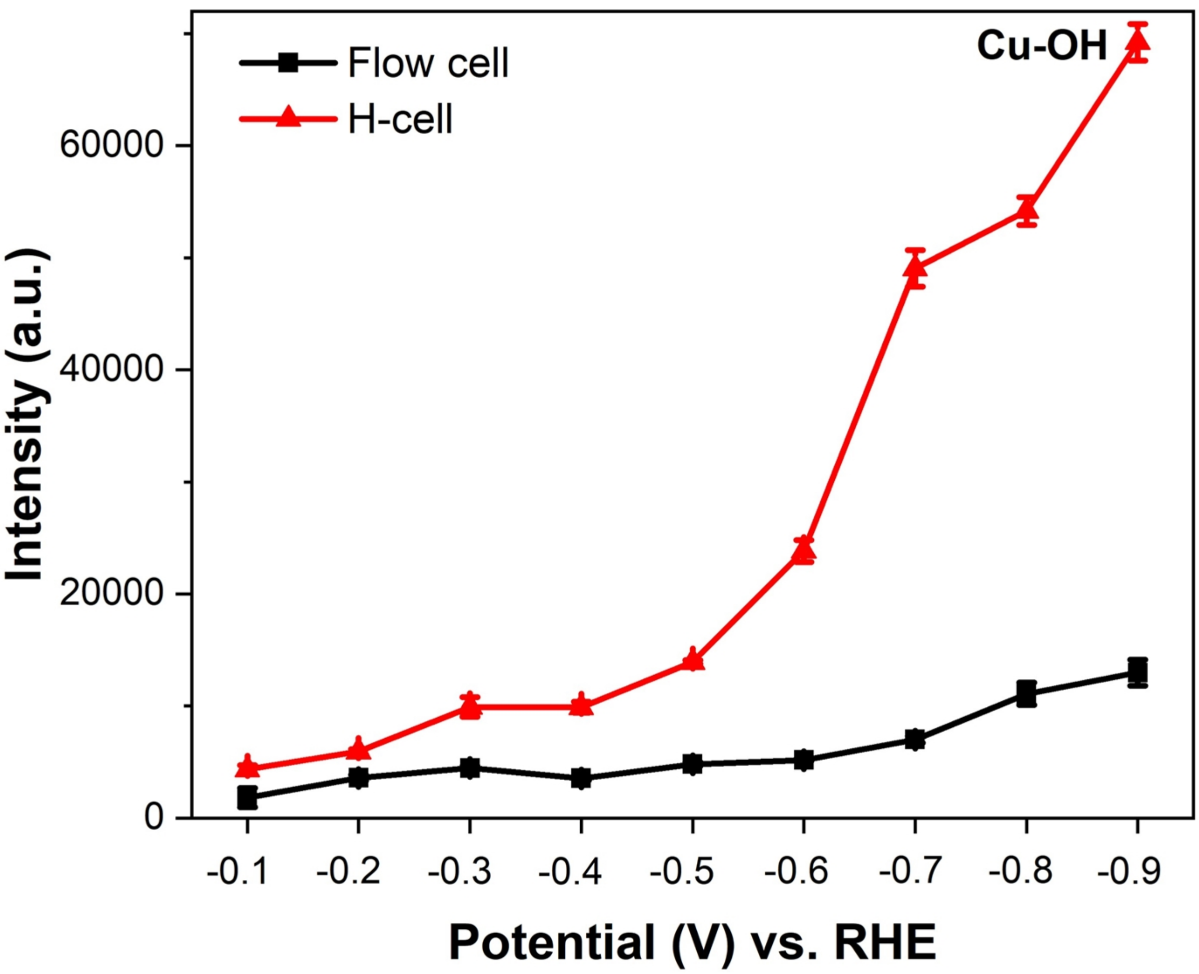

Supplementary Figure 20. Comparison of the intensity of Cu-OH band on Cu catalysts in CO-saturated 0.5 M KOH collected in the H-type spectroelectrochemical cell and the GDE-type spectroelectrochemical flow cell at various potentials. The error bars represent the standard deviation from at least three independent measurements.

Supplementary References

(1) Chernyshova, I. V.; Somasundaran, P.; Ponnurangam, S. On the origin of the elusive first intermediate of CO_2 electroreduction. *Proc. Natl. Acad. Sci. U.S.A.* **2018,** *115* (40), E9261-E9270.

(2) Moradzaman, M.; Mul, G. In Situ Raman Study of Potential-Dependent Surface Adsorbed Carbonate, CO, OH, and C Species on Cu Electrodes During Electrochemical Reduction of CO_2. *ChemElectroChem* **2021,** *8* (8), 1478-1485.

(3) Chang, X.; Zhao, Y.; Xu, B. pH Dependence of Cu Surface Speciation in the Electrochemical CO Reduction Reaction. *ACS Catal.* **2020,** *10* (23), 13737-13747.

(4) An, H.; Wu, L.; Mandemaker, L. D. B.; Yang, S.; de Ruiter, J.; Wijten, J. H. J.; Janssens, J. C. L.; Hartman, T.; van der Stam, W.; Weckhuysen, B. M. Sub-Second Time-Resolved Surface-Enhanced Raman Spectroscopy Reveals Dynamic CO Intermediates during Electrochemical CO_2 Reduction on Copper. *Angew. Chem. Int. Ed.* **2021,** *60* (30), 16576-16584.

(5) Yan, W.; Wu, T. T.; Liu, J.; Zheng, Z.; Ma, M. Mass Transport-Dependent C-C Bond Formation for CO Electroreduction with Alkali Cations. *J. Am. Chem. Soc.* **2025,** *147* (11), 9990-10001.

(6) Zhan, C.; Dattila, F.; Rettenmaier, C.; Bergmann, A.; Kühl, S.; García-Muelas, R.; López, N.; Cuenya, B. R. Revealing the CO Coverage-Driven C-C Coupling Mechanism for Electrochemical CO_2 Reduction on Cu_2O Nanocubes via Operando Raman Spectroscopy. *ACS Catal.* **2021,** *11* (13), 7694-7701.

(7) An, H. Y.; de Ruiter, J.; Wu, L. F.; Yang, S.; Meirer, F.; van der Stam, W.; Weckhuysen, B. M. Spatiotemporal Mapping of Local Heterogeneities during Electrochemical Carbon Dioxide Reduction. *JACS Au* **2023,** *3* (7), 1890-1901.
